# Cellooligomer/CELLOOLIGOMER RECEPTOR KINASE1 Signaling Exhibits Crosstalk with PAMP-Triggered Immune Responses and Sugar Metabolism in Arabidopsis Roots

**DOI:** 10.3390/ijms25063472

**Published:** 2024-03-19

**Authors:** Akanksha Gandhi, Michael Reichelt, Alexandra Furch, Axel Mithöfer, Ralf Oelmüller

**Affiliations:** 1 Matthias Schleiden Institute of Genetics, Bioinformatics and Molecular Botany, Department of Plant Physiology, Friedrich-Schiller-University Jena, Dornburger Str. 159, 07743 Jena, Germany; agandhi@ice.mpg.de (A.G.);; 2 Department of Biochemistry, Max-Planck-Institute for Chemical Ecology, Hans Knöll Str. 8, 07745 Jena, Germany; 3 Research Group Plant Defense Physiology, Max Planck Institute for Chemical Ecology, Hans Knöll Str. 8, 07745 Jena, Germany

**Keywords:** cellooligomer, CELLOOLIGOMER RECEPTOR KINASE1, BAK1, FERONIA, SWEET11, SWEET12, SUCROSE-PROTON SYMPORTER1

## Abstract

The degradation of cellulose generates cellooligomers, which function as damage-associated molecular patterns and activate immune and cell wall repair responses via the CELLOOLIGOMER RECEPTOR KINASE1 (CORK1). The most active cellooligomer for the induction of downstream responses is cellotriose, while cellobiose is around 100 times less effective. These short-chain cellooligomers are also metabolized after uptake into the cells. In this study, we demonstrate that CORK1 is mainly expressed in the vascular tissue of the upper, fully developed part of the roots. Cellooligomer/CORK1-induced responses interfere with chitin-triggered immune responses and are influenced by BRASSINOSTEROID INSENSITIVE 1-ASSOCIATED RECEPTOR KINASE1 and the receptor kinase FERONIA. The pathway also controls sugar transporter and metabolism genes and the phosphorylation state of these proteins. Furthermore, cellotriose-induced ROS production and *WRKY30*/*40* expression are controlled by the sugar transporters SUCROSE-PROTON SYMPORTER1, SUGARS WILL EVENTUALLY BE EXPORTED TRANSPORTER11 (SWEET11), and SWEET12. Our data demonstrate that cellooligomer/CORK1 signaling is integrated into the pattern recognition receptor network and coupled to the primary sugar metabolism in Arabidopsis roots.

## 1. Introduction

The plant cell wall is a natural barrier against abiotic and biotic stress [1,2,3]. Breakdown of the cell wall results in the apoplastic accumulation of oligosaccharides, which are recognized by cell surface receptors as damage-associated molecular patterns (DAMPs). The pattern recognition receptors (PRRs) for the breakdown products of pectin and oligogalacturonides are WALL-ASSOCIATED KINASES [4], and for cellooligomers derived from cellulose is the CELLOOLIGOMER RECEPTOR KINASE1 (CORK1)/IMPAIRED IN GLUCAN PERCEPTION1 (IGP1) [5,6]. The perception of mixed-linkage β-1,3/1,4-glucan oligosaccharides from the breakdown of some hemicelluloses involves IGP2/3 and IGP4 [6], CHITIN ELICITOR RECEPTOR KINASE1 (CERK1), and the LysM-containing receptor-like kinases 4 and 5 (LYK4 and LYK5) [7,8,9]. Breakdown of callose results in the accumulation of non-branched β-1,3-glucan oligosaccharides perceived by CERK1, LYK4, and LYK5 [7]. Xyloglucans from hemicellulose function as DAMPs in different plant systems; however, a receptor has not yet been identified [10]. Cellooligosaccharide perception is considered as an alarm system which informs the cell about the integrity of its wall [11]. Besides cell wall repair mechanisms, the responses triggered by cell wall degradation products overlap with those triggered by pathogen-associated molecular patterns (PAMPs) from pathogens [12]. Furthermore, cell wall degradation products are energy sources for the plant cells as well as for their interacting microbes [13].

Cellulose breakdown generates cellooligomers, of which those with 2–7 glucose moieties induce cytoplasmic Ca^2+^ ([Ca^2+^]_cyt_) elevation, reactive oxygen species (ROS) generation, plasma membrane depolarization, alterations in the phosphorylation patterns of numerous proteins, and defense gene activation via MAPK3/6 signaling [5,6,14,15]. Among the rapidly stimulated genes which respond to cellooligomer application to Arabidopsis roots are the *WRKY30* and *WRKY40* transcription factors [5]. Cellotriose (CT) is the most active cellooligomer [14], and CT and cellopentaose bind to the ectodomain of CORK1/IGP1, a leucine-rich repeat (LRR)-malectin receptor kinase [6]. Furthermore, the CYTOPLASMIC Ca^2+^ ELEVATION MUTANT (CYCAM) protein, which is the only poly(A)-ribonuclease in Arabidopsis, is required for the CT-induced responses in Arabidopsis [14]. Among the rapidly phosphorylated proteins upon CT application to wild-type roots, but not *cork1* roots, are BRASSINOSTEROID INSENSITIVE 1-ASSOCIATED RECEPTOR KINASE1 (BAK1) and FERONIA (FER) [5], suggesting crosstalk between CORK1, BAK1, and FER signaling. BAK1 is the coreceptor of many PRRs, including BRASSINOSTEROID INSENSITIVE1 (BRI1), FLAGELLIN-SENSITIVE2 (FLS2) [16,17,18,19], EF-TU RECEPTOR, and PEP1 RECEPTOR1/2 [20,21,22,23,24,25], and it interacts with ERECTA and ERECTA-LIKE1, which recognize the peptides EPIDERMAL PATTERNING FACTOR1 (EPF1) and EPF2 involved in stomatal development and patterning [26]. BAK1 also participates in ROOT GROWTH FACTOR RECEPTOR 1 (RGFR1) and RGFR5 signaling, which perceive ROOT GROWTH FACTOR peptides and mediate root meristem development [27,28]. FER is involved in growth and development, biotic and abiotic stress responses, and reproduction [29,30]. Furthermore, since cellooligomer-induced responses overlap with those induced by chitin [14], we compared them with responses induced by chitin to investigate whether there was crosstalk between the signaling pathways in Arabidopsis roots. Networks between PRRs, coreceptors, and associated signaling components have been well investigated [31,32]; however, whether there is crosstalk between the cellooligomer/CORK1 pathway and other PRRs has not yet been studied. 

Cellulose degradation by plant and microbial enzymes generates short-chain cellooligomers and ultimately glucose, which are both taken up by the cell. It is believed that most of the degradation products are utilized for cell wall regeneration [33]; however, it is also possible that some of them are integrated into the primary sugar metabolism and serve as an energy source. The availability of reduced carbon also impacts the growth of the symbiotic and pathogenic microorganism associated with the hosts [13,33]. SUGAR TRANSPORTER PROTEINs (STPs) are proton-coupled symporters responsible for the uptake of glucose from the apoplast into plant cells, where they function as key signaling molecules and play a vital role in plant responses to stressors such as dehydration and prevalent fungal infections [34,35]. The SUGARS WILL EVENTUALLY BE EXPORTED TRANSPORTERS (SWEETs) redirect sugars in the plant tissues and deprive colonizing microorganisms of reduced carbon, thereby interfering with the innate immune responses [34,36]. Multiple studies suggest that there is crosstalk between the oligosaccharide/DAMP-triggered cell wall surveillance systems and the primary sugar metabolism in the plant cell. STP13 affects glucose transport, activates biotic and abiotic stress signaling, and confers basal resistance against pathogens [37,38,39]. Trehalose-6-phosphate is a central regulator of sugar metabolism in plants and modulates basal defense responses [40,41,42]. The systemic defense signaling molecules AZELAIC ACID INDUCED1 and glycerol-3-phosphate are connected to sugar signaling [43,44]. AZELAIC ACID INDUCED1 is a protein which is required for azelaic acid transport, or the transport of an azelaic acid-induced signal to activate immune responses in systemic tissues [43]. Moreover, Kohorn et al. [45] have shown that cell wall sensing via pectin breakdown regulates the vacuolar invertase involved in the solute sugar metabolism. The cellulose breakdown product cellobiose (CB) stimulates the primary sugar metabolism and evokes innate immune responses [46]. The stimulation of immune responses by cellooligomers may result in a shift of the growth/defense balance towards defense, which not only inhibits growth and activates defense gene expression but also affects the amino acid profile [47,48,49]. Under unfavorable conditions, specific stress-related amino acids accumulate for counteracting stress situations, as well as redox, metabolic, and osmotic imbalances [49]. Here, we present evidence that cellooligomer/CORK1 signaling controls genes involved in sugar distribution and metabolism, and that sugar transport mutants are impaired in immune responses elicited by cellooligomer/CORK1 signaling. Our study demonstrates that cellooligomer/CORK1 signaling is integrated into the PRR network that is active in Arabidopsis roots and coupled to the primary sugar metabolism.

## 2. Results

### 2.1. CORK1 Localization

**Figure 1:**
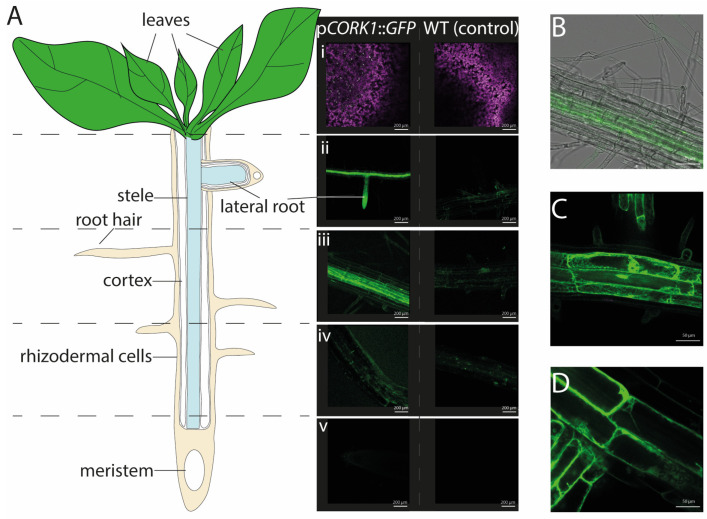
(**A**) The cartoon depicts root developmental regions and, in parallel, representative fluorescence images from p*CORK1*::*GFP* expression in 14-day-old transgenic Arabidopsis seedlings, in comparison to the wild-type (control) seedlings. The promoter activity was not detectable in the aerial parts of the plant (**i**). The roots exhibit tissue-specific *CORK1* expression patterns. A high GFP fluorescence is observed in the lateral roots (**ii**), root stele (**iii**), and cortical tissue while barely any fluorescence can be detected in the rhizodermal cells layer (**iv**). No fluorescence was found in the meristematic zone and root tip (**v**). (**B**) Confocal microscopy of 14-day-old transgenic Arabidopsis seedlings illustrates GFP fluorescence driven by the *CORK1* promoter in the early root differentiation zone. (**C**,**D**) These panels show the subcellular location of the GFP fluorescence in the cytoplasm. Representative results were observed for six plants from three independent transformants.

Expression profiles [5] and the response patterns of Arabidopsis seedlings to cellooligomer treatments [5,14] suggest that CORK1 is mainly active in the roots. In this study, confocal laser scanning microscopy of Arabidopsis seedlings expressing a *CORK1* promoter::*GFP* fusion construct confirmed that GFP signals were preferentially detectable in the roots and barely in the leaf and stem tissues (Figure 1A). The highest fluorescence was found in the upper part of the main roots, the root stele, the cortical tissue, as well as in the lateral roots emerging from them. Interestingly, the tips of these lateral roots emitted quite high fluorescence (Figure 1A(ii)). Furthermore, the GFP activity was mainly detectable in the vascular tissue (Figure 1A,B), not in the rhizodermal, subepidermal, or epidermal cell layers, nor in the root hairs (Figure 1B). When sections from the lower part of the roots were analyzed, the activity appeared to decline gradually towards the root tip. GFP signals were still measurable in the elongation zone, while no fluorescence was visible in the root tip (Figure 1A). At the cellular level, the fluorescence signals from the soluble GFP were visible in the cytoplasm between the large vacuole and the plasma membrane, often enriched around the nucleus (Figure 1C,D). Taken together, the *CORK1* promoter appears to be mainly active in the vascular tissue of the upper part of the roots. This is consistent with RT-qPCR analyses which show that the *CORK1* transcript level is significantly higher in roots than in shoots of 14-day-old Arabidopsis seedlings (roots: 22.2 ± 0.5; shoots 3.1 ± 0.3 ****; n = 9).

### 2.2. CORK1 Expression Is Not Stimulated by Isoxaben (ISX)

**Figure 2:**
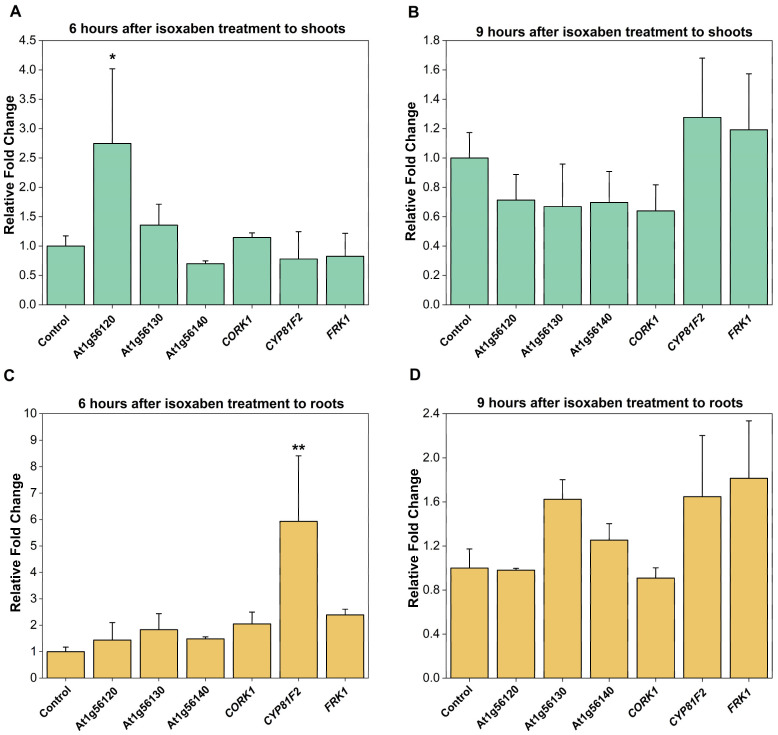
Expression of receptor kinase genes in 14-day-old Col-0 plants determined by qRT-PCR after application of 0.6 µM ISX to the roots. Shoots (**A**,**B**) and roots (**C**,**D**) were separately harvested 6 h (**A**,**C**) or 9 h (**B**,**D**) after ISX application. *FRK1* (encoding FLG22-INDUCED RECEPTOR-LIKE KINASE1, At2g19190) and *CYP81F2* (encoding a P450 enzyme involved in glucosinolate metabolism, At5g57220) are immune marker genes and were used as controls. Depicted is the fold change in expression relative to the DMSO control without ISX. Error bars represent the standard error of three biological replicates. Asterisks indicate a statistically significant difference relative to the control as determined by one-way ANOVA followed by a Holm–Sidak post-hoc test (* *p* ≤ 0.05; ** *p* ≤ 0.01).

CORK1, and the phylogenetically related At1g56130 (IGP2/3) and At1g56140 (IGP4) have been shown to be involved in glycan perception, and knock-out or point mutation mutants are defective in the activation of pattern-triggered immunity mediated by CT (CORK1) or mixed-linked β-1,3/β-1,4-glucan oligosaccharides (At1g56130 and At1g56140), but not by fungal chitin oligosaccharides [5,6]. When ISX, which inhibits cellulose biosynthesis and thus impairs proper de novo cell wall biosynthesis [50], was applied to Arabidopsis roots for 6 or 9 h, we observed no effect on the expression of *CORK1* and the related malectin-containing receptor kinase genes *At1g56130* and *At1g56140* in the roots (Figure 2). Only the uncharacterized *At1g56120* gene, which is closely related to the *CORK1*, IGP2/*3*, and *IGP4* genes, and also a member of the LRR-malectin domain-receptor kinase gene subfamily [6,10], was significantly upregulated in the shoots (cf. Section 3). These results suggest that the *CORK1*, *At1g56130*, and *At1g56140* genes do not respond to the inhibition of cellulose biosynthesis (cf. Section 3). 

### 2.3. Activation of CORK1-Dependent Responses by Disaccharides

**Figure 3:**
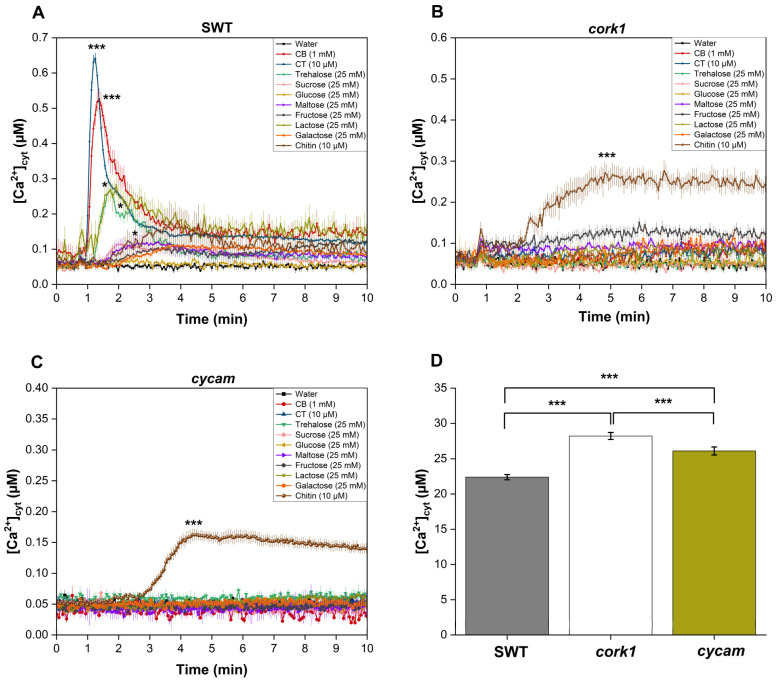
[Ca^2+^]_cyt_ elevation in the roots of 14-day-old segregating wild-type (SWT) (**A**) and *cork1* (**B**) seedlings expressing aequorin after application of different sugars. [Ca^2+^]_cyt_ elevation was observed after application of CT (10 µM), CB (1 mM), and trehalose or lactose (25 mM each) in the SWT but not in the *cork1* and *cycam* (**C**) mutants. Chitin (10 µM) was used as a control and the *cork1* and *cycam* mutations did not affect its response. (**D**) Comparison of the total [Ca^2+^]_cyt_ levels (area under the curve) upon chitin application to the roots of SWT, *cork1*, and *cycam* seedlings. Total [Ca^2+^]_cyt_ levels were significantly higher in *cork1* compared to SWT and *cycam*. Error bars represent the standard error from eight seedlings. Data are based on three independent experiments. Statistical significance at the peak value was determined by Tukey’s HSD test (* *p* ≤0.05; *** *p* ≤ 0.001). Asterisks indicate significant differences compared to the water treatment (**A**–**C**).

**Figure 4:**
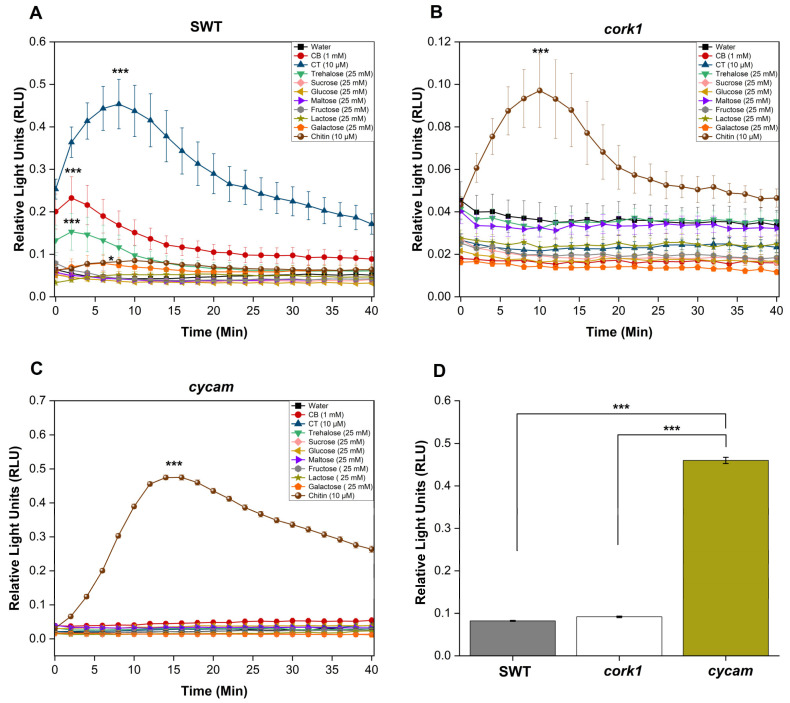
The *cork1* and *cycam* mutants failed to induce ROS production after the application of different sugars. CT (10 µM), CB (1 mM), or trehalose (25 mM) trigger ROS production in the root tissue of the segregating wild-type (SWT) (**A**) but not in the *cork1* (**B**) and *cycam* (**C**) seedlings. ROS production after the application of chitin was not affected by the mutations. (**D**) Comparison of the maximum ROS production in response to chitin application at the peak time in the roots of SWT, *cork1*, and *cycam* seedlings. ROS production was significantly higher in *cycam* roots compared to SWT and *cork1* roots. Error bars represent SE from eight seedlings for each treatment. Statistically significant differences between CT, CB, trehalose, and chitin versus water treatment at the peak value were determined by Tukey’s HSD (* *p* ≤ 0.05; *** *p* < 0.001). The experiment was repeated three times with similar results.

**Figure 5:**
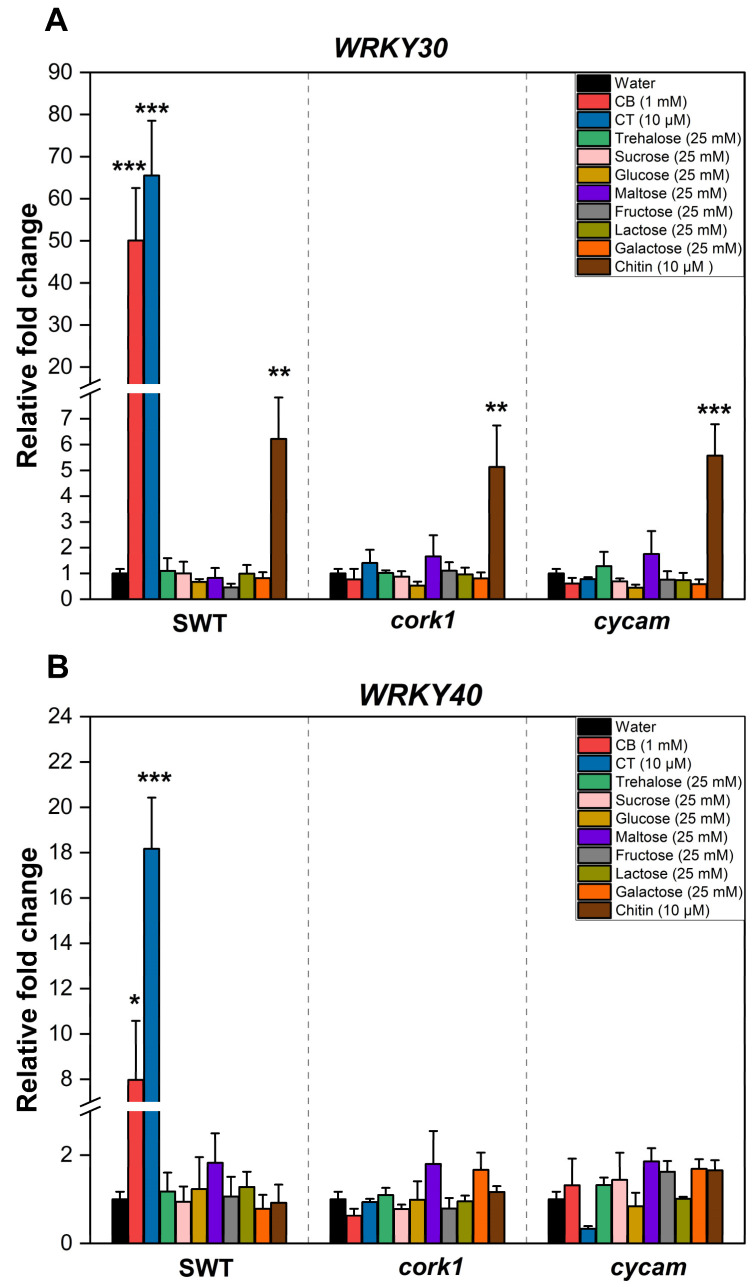
Upregulation of (**A**) *WRKY30* and (**B**) *WRKY40* mRNA levels in the roots was impaired in *cork1* and *cycam* mutants upon treatment with either 10 μM CT or 1 mM CB for one hour, but not in the segregating wild-type. The expression of *WRKY30* was not affected by these mutations upon application of 10 µM chitin. Values were normalized to water treatment on the same genotype. Error bars represent SE from three independent biological replicates, each with twenty seedlings. Statistical significance between water and other treatments within the same genotype was accessed by one-way ANOVA and Tukey’s HSD. Asterisks indicate statistically significant differences between water treatment and treatment with the indicated chemical within the same genotype (* *p* ≤ 0.05; ** *p* ≤ 0.01; *** *p* ≤ 0.001).

Previous studies have suggested that cellooligomers with 2–7 glucose moieties are perceived by root cells [14]. CB was intensively studied by Souza et al. [51]. CT, the most active cellooligomer, activated various CORK1-dependent cellular responses [5]. Martin-Dacal et al. [6] demonstrated the binding of CT and cellopentaose to the ectodomain of the LRR-malectin receptor kinase CORK1/IGP1. The mammalian malectin proteins recognize the disaccharides maltose and, to a lesser extent, nigerose [52]. Therefore, we tested whether disaccharides other than CB induce cellular responses in Arabidopsis roots of the segregating wild-type (SWT), but not *cork1* mutant seedlings. CORK1-dependent [Ca^2+^]_cyt_ elevation (Figure 3), ROS production (Figure 4), and *WRKY30* and *WRKY40* expression (Figure 5) were stimulated by 10 µM CT. *WRKY30* and *WRKY40* have been previously identified to be strongly upregulated after CT application to Arabidopsis roots [5,51]. Therefore, the two genes are ideal for assaying cellooligomer-induced changes in gene expression. CORK1-dependent responses to CB required at least 1 mM, while those induced by lactose and trehalose required 25 mM. An amount of 25 mM sucrose, glucose, maltose, fructose, nigerose, or galactose did not induce the three cellular responses in a CORK1-dependent manner. Furthermore, the previously characterized *cycam* mutant, which is impaired in the poly(A)-ribonuclease in Arabidopsis [14], also failed to induce [Ca^2+^]_cyt_ elevation (Figure 3), ROS production (Figure 4), and *WRKY30*/*40* expression (Figure 5) in response to CT (10 µM) [14], CB (1 mM), and lactose and trehalose (25 mM). Although the high disaccharide concentrations are unphysiological, the differences in the responses of the SWT and *cork1*/*cycam* seedlings observed for lactose and trehalose demonstrated a requirement of CORK1 and the poly(A)ribonuclease for the induction of the responses. Apparently, CORK1 and the poly(A) ribonuclease are common signaling components for the responses induced by CT and the three disaccharides (cf. Section 3).

### 2.4. Crosstalk between CT and Chitin Signaling

Chitin induces [Ca^2+^]_cyt_ elevation (Figure 3), ROS production (Figure 4), and *WRKY30*/*40* expression (Figure 5) in the SWT, *cork1*, and *cycam* mutants; however, we observed marked differences. The [Ca^2+^]_cyt_ elevation in response to chitin application was significantly lower in the SWT compared to both mutants, indicating that CORK1 and CYCAM inhibit chitin-induced [Ca^2+^]_cyt_ elevation (Figure 3D). Moreover, ROS production in response to chitin application was comparable for the SWT and the *cork1* mutant but significantly higher in the *cycam* mutant (Figure 4D). This suggests crosstalk between the CT- and chitin-triggered signaling pathways, and that CORK1 and CYCAM have different effects on the two responses (cf. Section 3). No significant differences were observed for the chitin-induced *WRKY30* and *WRKY40* expression (Figure 5). Thus, the increase in [Ca^2+^]_cyt_ elevation does not correlate quantitatively with the regulation of the defense genes.

### 2.5. BAK1 Inhibits CT-Induced ROS Production in Arabidopsis Roots

**Figure 6:**
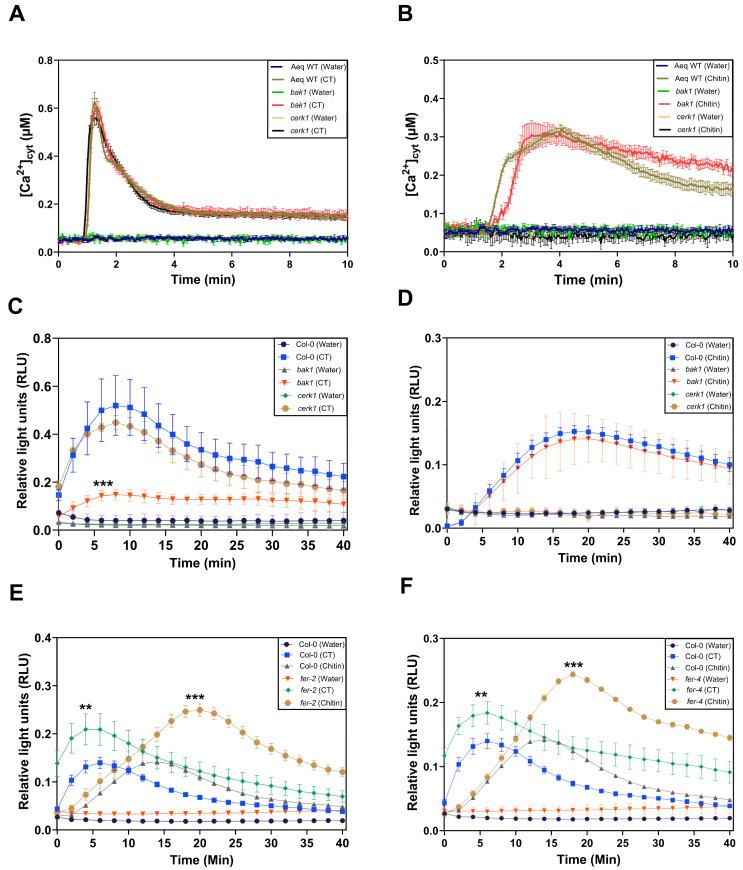
BAK1 inhibits CT-induced ROS production but not CT-induced [Ca^2+^]_cyt_ elevation in Arabidopsis roots. [Ca^2+^]_cyt_ elevation in roots of the aequorin wild-type, aequorin *bak1*, and aequorin *cerk1* seedlings after application of 10 μM CT (**A**) or 10 μM chitin (**B**). Total ROS production over a period of 40 min represented as relative light units (RLUs) in the roots of the same wild-type, *bak1*, and *cerk1* seedlings after elicitation with 10 μM CT (**C**) or 10 μM chitin (**D**). ROS production is significantly higher in *fer-2* (**E**) and *fer-4* (**F**) mutants compared to wild-type (Col-0) seedlings after the application of 10 μM CT or 10 μM chitin. Error bars represent SE from eight seedlings for each treatment. Asterisks indicate statistically significant differences at the peak value compared to the Col-0 or aequorin wild-type control as determined by one-way ANOVA coupled to Tukey’s HSD (** *p* ≤ 0.01; *** *p* ≤ 0.001). The experiment was repeated three times with similar results.

The application of CT to Arabidopsis roots leads to the rapid phosphorylation of BAK1, and the response requires CORK1 [5]. Since BAK1 is a coreceptor of many PRRs (cf. Section 1), we tested whether BAK1 is involved in CT-induced [Ca^2+^]_cyt_ elevation or ROS production in Arabidopsis roots. We compared CT-induced responses in wild-type and *bak1* roots (Figure 6A–D). Chitin was used as a control. [Ca^2+^]_cyt_ elevation in response to CT or chitin application was not affected by the *bak1* mutation (Figure 6A,B), while ROS production was significantly lower after CT application, but not chitin application, to the *bak1* mutant, when compared to the wild-type and *cerk1* roots (Figure 6C,D). This indicates that BAK1 affects CT-, but not chitin-induced ROS production, while [Ca^2+^]_cyt_ elevation in response to both D/PAMPs is not affected by BAK1. CT-induced [Ca^2+^]_cyt_ elevation and ROS production are not affected in the *cerk1* mutant (Figure 6A,C).

### 2.6. FER Restricts CT- and Chitin-Induced ROS Production in Arabidopsis Roots

The malectin domain containing receptor kinase FER is rapidly phosphorylated in SWT roots, but not *cork1* roots, exposed to CT [5]. ROS production in two FER mutants, *fer-2* (Figure 6E) and *fer-4* (Figure 6F), in response to 10 μM CT or 10 μM chitin application, was significantly higher in comparison to the SWT seedlings. Since no significant differences for CT- or chitin-induced *WRKY30* and *WRKY40* expression were observed for the wild-type and the two *fer* mutants (Supplementary Figure S1), the FER-mediated restriction of ROS production in response to the two D/PAMPs might be caused by the direct inhibition of the NADPH oxidase activity (cf. Section 3).

### 2.7. CT Alters the Expression and Phosphorylation Patterns of Sugar Transport and Metabolism Proteins

The inspection of expression profiles of CT-responsive genes in Arabidopsis roots [5] uncovered that the mRNA levels for sugar-related genes, in particular sugar transporter genes, are either up- or downregulated in the SWT, but not in the *cork1* mutant, 1 h after CT application. For instance, genes for EARLY RESPONSE TO DEHYDRATION6 (ERD6) and POLYOL/MONOSACCHARIDE TRANSPORTER6 (PMT6) are upregulated while those for the SUCROSE-PROTON SYMPORTER2 (SUC2), the SUGAR TRANSPORT PROTEIN1 (STP1) and STP4, ERD6-LIKE1, and the SUCROSE TRANSPORTER2 (SUT2) are downregulated in SWT roots, but not—or to a significantly lesser extent—in the *cork1* mutant roots (Supplementary Table S1). Furthermore, the phosphorylation patterns of proteins involved in sugar transport and metabolism also changed in response to CT application in the SWT, but not in the *cork1* roots. This was observed for the sugar transporters ERD6, SWEET12, STP7, and MONOSACCHARIDE-SENSING1 (MSSP1), but also for enzymes involved in the sugar metabolism (SUCROSE-PHOSPHATE SYNTHASE1 (SPS1) and SPS2; SUCROSE SYNTHASE6 (SUS6)) and for the SUCROSE NONFERMENTING 4-LIKE PROTEIN (SNF4) which regulates the cell response to glucose starvation (TAIR) (Supplementary Table S2). Since none of these proteins are directly involved in cellulose biosynthesis, we hypothesized that CT/CORK1 signaling exhibits crosstalk with the primary sugar metabolism/transport. 

### 2.8. Sucrose Stimulates SUC1, SWEET11, and SWEET12 Expression in SWT Roots and Inhibits Their Expression in cork1 Roots

**Figure 7:**
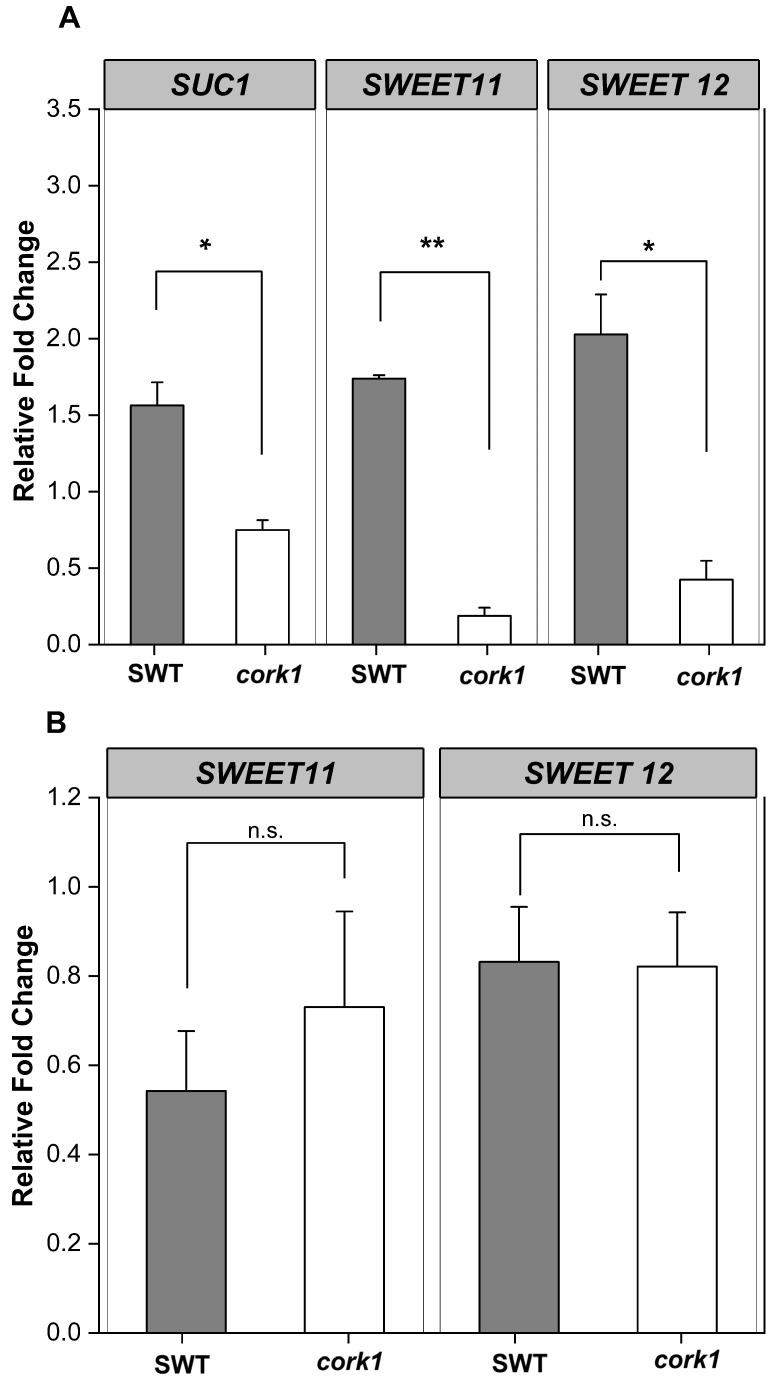
Sucrose (**A**), but not glucose (**B**), stimulates the expression of the sugar transporter genes *SUC1*, *SWEET 11*, and *SWEET 12* in the roots of the segregating wild-type seedlings, but not in the *cork1* mutant seedlings, 1 h after sugar application. Values were normalized to water treatment on the same genotype. Error bars represent SE from at least three independent biological replicates, each with twenty seedlings. Statistical significance was determined by a two-tailed Student’s *t*-test (n.s., no significant differences, * *p* ≤ 0.05; ** *p* ≤ 0.01). Regulation of *SUC1* by glucose was omitted because *SUC1* transports only sucrose.

To further investigate whether CORK1 is involved in the sugar metabolism/transport, we investigated the expression of *SUC1*, *SWEET11*, and *SWEET12* in the roots of SWT and *cork1* seedlings. These genes were chosen because they are expressed in roots and involved in sucrose distribution in the root tissue (cf. Section 3). qPCR analyses for *SUC1*, *SWEET11*, and *SWEET12* demonstrate that sucrose, but not glucose, stimulates their expression in SWT roots within 1 h, whereas their expression is inhibited in *cork1* roots (Figure 7). The requirement of CORK1 for the stimulatory sucrose effect, combined with the strong inhibition of their expression in the *cork1* mutant line, provides further evidence for crosstalk between CORK1 signaling and the sugar transporters (cf. Section 3).

### 2.9. SUC1, SWEET11, and SWEET12 Control CT-Induced ROS Production and WRKY30/40 Expression

**Figure 8:**
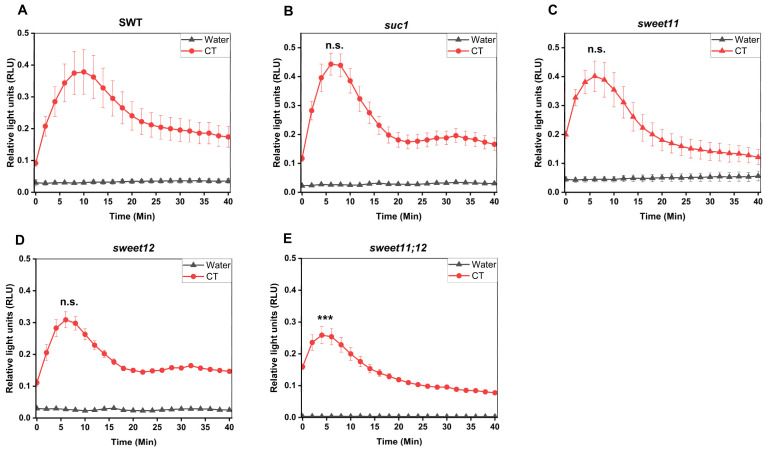

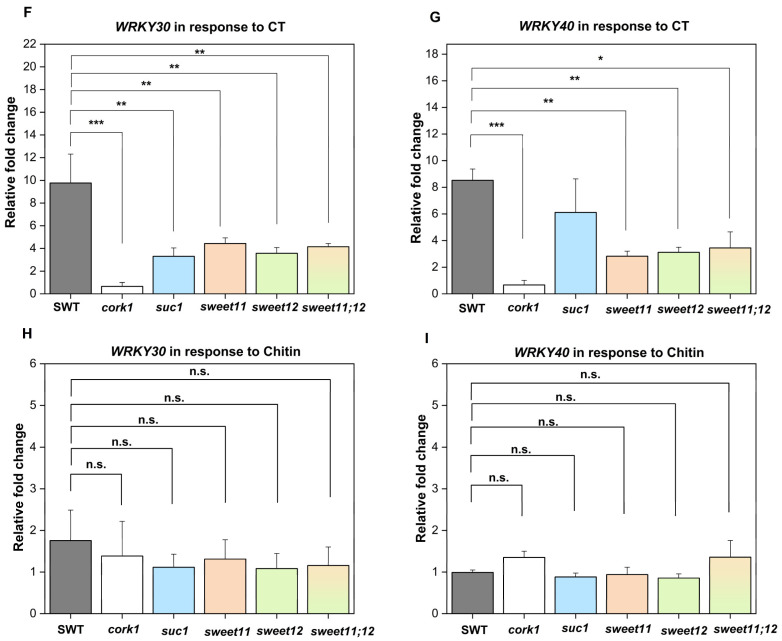
SUC1, SWEET11, and SWEET12 control CT responses in Arabidopsis roots. (**A**–**E**) ROS production in the roots of the 14-day-old (**A**) segregating wild-type (SWT), (**B**) *suc1*, (**C**) *sweet11*, (**D**) *sweet12*, *(***E**) and *sweet11;12* double mutants (**F**) upon application of 10 μM CT. ROS generation was significantly reduced in the *sweet11;12* double mutant as compared to the SWT, as indicated by asterisks. Error bars represent SEM from eight seedlings for each treatment. One-way ANOVA and Tukey’s HSD were used to compare differences between the SWT and the other genotypes within each treatment (*** *p* ≤ 0.001; n.s., not significant). The experiment was repeated three times with similar results. (**F**–**I**) Eleven-day-old seedlings were treated with either 10 μM CT or chitin for 1 h, and the expression of *WRKY30* and *WRKY40* was determined by qRT-PCR. The graphs show the average data of three biological replicates. Expression of *WRKY30* (**F**) and *WRKY40* (**G**) was significantly reduced in *cork1*, *suc1* (only for *WRKY30*), *sweet11*, *sweet12*, and *sweet11;12* mutants upon CT application. (**H**,**I**) No significant regulation of the two genes was detected in response to chitin application in comparison to the SWT. Error bars indicate SEM and statistical comparisons were made using one-way ANOVA with Holm–Sidak post hoc analysis (* *p* ≤ 0.05; ** *p* ≤ 0.01; *** *p* ≤ 0.001).

To test whether CT-induced responses are dependent on the sugar transporters SUC1, SWEET11, and SWEET12, we compared ROS production as well as *WRKY30/40* gene expression in SWT seedlings and *suc1*, *sweet11*, *sweet12*, and *sweet11;12* double knock-out lines. Figure 8A–E demonstrates that ROS production in response to CT application in the *sweet11;12* double knock-out line was significantly reduced compared to the wild-type and all single knock-out lines. Furthermore, stimulation of *WRKY30* and *WRKY40* expression by CT was significantly inhibited in all sugar mutants, while no significant differences were observed for their regulation in response to chitin (Figure 8F–I). This demonstrates that knocking out these sugar transporters represses CT-induced *WRKY30/40* expression, while ROS production is less affected in the sugar transporter mutants. In conclusion, Figure 7 and Figure 8 demonstrate that CT/CORK1 influences the expression of sugar transporter genes and sugar transporters influence CT/CORK1-induced responses.

### 2.10. Long- and Short-Term Exposure of SWT and cork1 Seedlings to CT or CB

The activation of immune and stress responses by cellooligomers [5,6,14] suggests that the growth/stress response balance might be altered. In addition, in Arabidopsis roots many CT-responsive genes are also induced by PAMPs, such as chitin [5,14], which restricts growth in favor of defense activation. When nine-day-old SWT and *cork1* seedlings were either transferred to plant nutrient media (PNM) [53] with different CB concentrations (0–100 mM), or to PNM media with 10 µM CT for an additional 6 days, we observed that the root weights, but not fresh shoot weights, of the SWT seedlings were reduced after the application of 10 µM CT or CB concentrations > 30 mM, in contrast to the *cork1* seedlings where this reduction was not observed (Supplementary Figures S1 and S2). Sugar, glucosinolate, and phytohormone profiles were not affected by these treatments (Supplementary Figures S1 and S2) (cf. Section 3). This suggests that the inhibition of root growth by long-term exposure to cellooligomers requires CORK1.

**Figure 9:**
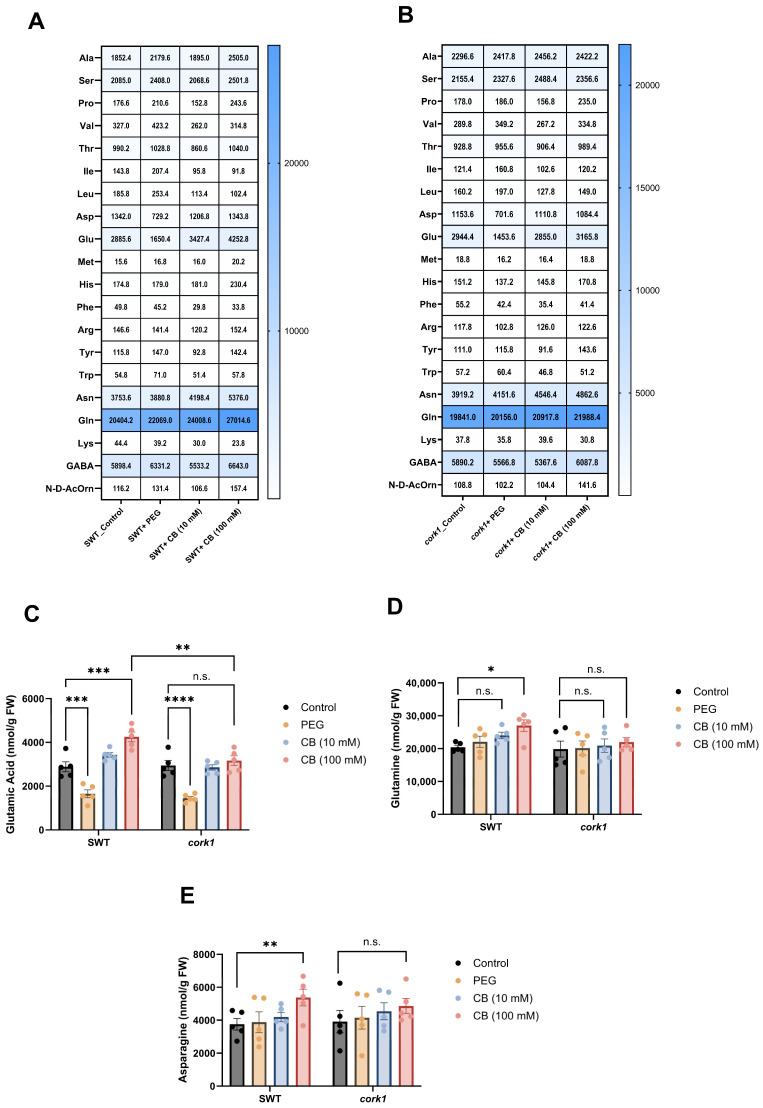
Effect of CB treatment (for 6 h) on amino acid levels (nmol/g FW) in the roots of segregating wild-type (SWT)and *cork1* seedlings. The heatmap displays a quantitative analysis of amino acids in the roots of SWT (**A**) and *cork1* (**B**) seedlings with or without CB. The SWT and *cork1* plants were pre-grown on half Murashige and Skoog media and then transferred to plant nutrient media (PNM) with different treatments (no treatment as control; 10 mM CB; 100 mM CB; 15 mM PEG 6000) for 6 h. PEG was used to eliminate the effect of osmotic shock. Amino acids were measured in 14-day-old plants (FW, fresh weight). The content of glutamic acid (**C**), glutamine (**D**), and asparagine (**E**) in the roots of the SWT and *cork1* after exposing them to 10 or 100 mM of CB for 6 h, is shown. Statistical significance was tested with two-way ANOVA using Tukey’s multiple comparison test (* *p* < 0.05; ** *p* < 0.01; *** *p* < 0.001; **** *p* < 0.0001; n.s., not significant). The data show the means from five independent experiments (n = 48–60 plants per treatment). Error bars represent SEM.

To test whether the growth inhibition by long-term exposure of the seedlings to CB might be caused by changes in the cellular metabolite profiles, we exposed 14-day-old SWT and *cork1* seedlings to 0, 10, and 100 mM CB for 6 h and measured the amino acid, glucosinolate, sugar, and phytohormone profiles in the roots. The rationale behind this experiment was that changes in these metabolite profiles are expected to occur early after the application of the cellooligomers and are no longer detectable after longer periods due to compensatory mechanisms in the plants (Supplementary Figure S2). Furthermore, CB can be metabolized faster by the roots than CT. PEG 6000 was used as a control to eliminate the effect of osmotic stress on the downstream responses caused by the highest CB concentration (100 mM). The comparison of the amino acid profiles of the SWT and *cork1* roots exposed to 100 mM CB or water uncovered that glutamic acid, glutamine, and asparagine contents were significantly higher in CB-treated SWT plants (Figure 9). Since this was not observed in the *cork1* roots after 100 mM CB treatment, the higher levels of these three amino acids should be caused by CB-mediated CORK1 activation (cf. Section 3). Moreover, the glutamic acid content in the CB-treated SWT roots was significantly higher than in the *cork1* roots.

**Figure 10:**
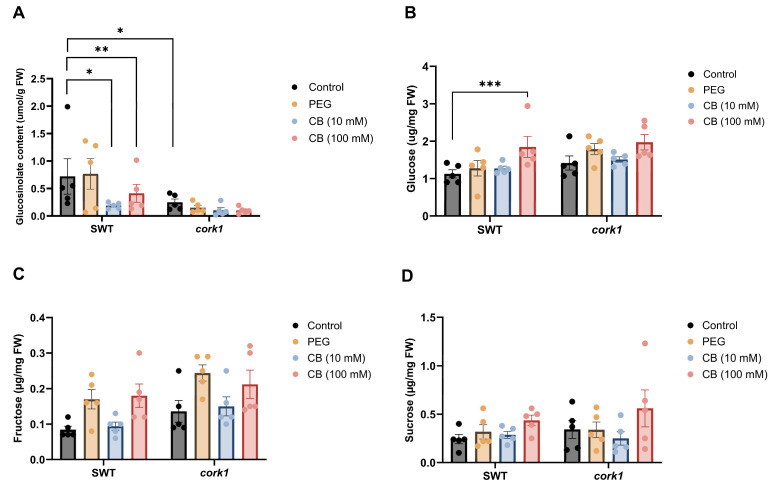
Accumulation of total glucosinolates and total soluble sugars in the roots of the 14-day-old segregating wild-type (SWT) and *cork1* plants 6 h after CB treatment. The SWT and *cork1* plants were pre-grown on half Murashige and Skoog media and then transferred to plant nutrient media (PNM) with different treatments (no treatment as control; 10 mM CB; 100 mM CB; 15 mM PEG 6000). PEG was used to eliminate the effect of osmotic shock. (**A**) Total glucosinolate content (µmol/g FW) in the SWT roots was significantly reduced upon exposure to media with 10 or 100 mM CB, while it was not affected by CB application in the roots of *cork1* (FW, fresh weight). The *cork1* control plants have significantly lower glucosinolate levels as compared to the SWT plants. (**B**) The glucose content (µg/mg FW) was significantly increased in the SWT roots but not in the *cork1* roots upon 100 mM CB treatment. (**C**) Fructose (µg/mg FW) and (**D**) sucrose (µg/mg FW) levels showed no significant differences between the SWT and *cork1* roots. The data correspond to means (±SEM) of five independent replicates (n = 48–60 plants per treatment). Asterisks represent significant differences between treatments according to two-way ANOVA with Tukey’s multiple comparison test (data were transformed when needed; * *p* < 0.05; ** *p* < 0.01; *** *p* < 0.001).

Interestingly, the total glucosinolate content in the SWT roots was significantly reduced upon exposure to 10 and 100 mM CB, while it was not affected by the CB treatments in the *cork1* roots (Figure 10A). However, the *cork1* roots which were not treated with CB contained already significantly lower glucosinolate levels than the SWT roots. This suggests that the restriction of glucosinolate accumulation in the SWT occurs via CB. The lower level of the secondary metabolites in the *cork1* roots is independent of CB and points to a CB-independent role of CORK1 in promoting glucosinolate biosynthesis. 

While no significant differences were observed in the fructose and sucrose levels in the CB-treated or untreated SWT and *cork1* roots (Figure 10C,D), the glucose level was significantly higher in the SWT roots than in the *cork1* roots upon 100 mM CB treatment (Figure 10B). 

**Figure 11:**
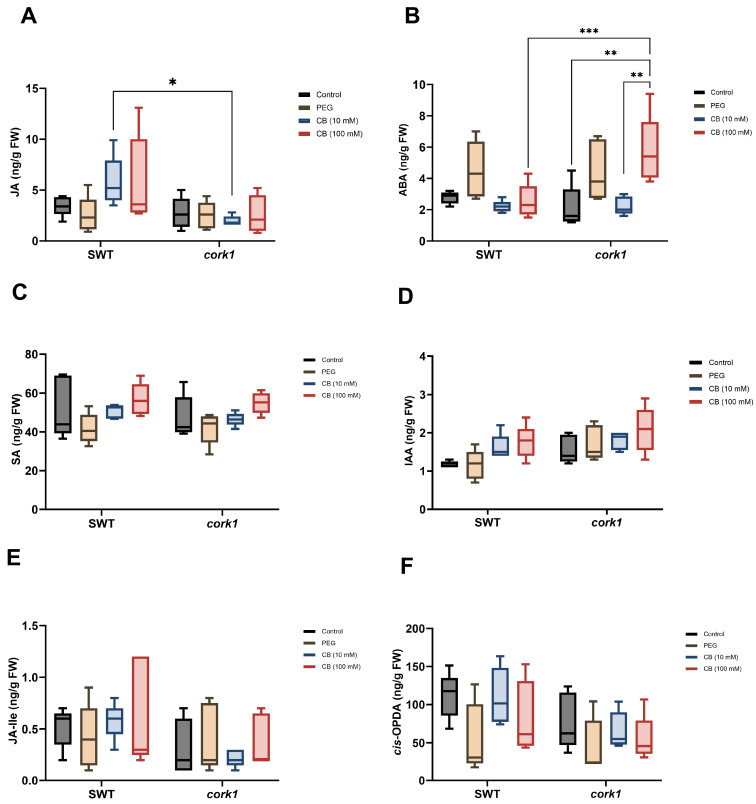
Phytohormone levels (ng/g FW) in the segregating wild-type (SWT) and *cork1* roots 6 h after treatment with 10 or 100 mM CB. The amounts of (**A**) JA, (**B**) ABA, (**C**) SA (salicylic acid), (**D**) IAA (indole-acetic acid), (**E**) the JA bioactive form JA-lle (JA-isoleucine), and (**F**) the JA precursor, *cis*-OPDA (*cis*-(+)-12-oxo-phytodienoic acid), were measured in the roots of 14-day-old Arabidopsis plants (FW, fresh weight). The SWT and *cork1* plants were pre-grown on half Murashige and Skoog media and then transferred to plant nutrient media (PNM) for 6 h with different treatments (no treatment as control; 10 mM CB; 100 mM CB; 15 mM PEG 6000). PEG was used to eliminate the effect of osmotic shock. Asterisks represent significant differences between treatments according to two-way ANOVA with Tukey’s multiple comparison test (* *p* < 0.05; ** *p* < 0.01; *** *p* < 0.001). The data show the means (±SE) of five replicates, each of which consisted of 48–60 seedlings.

Finally, among the analyzed phytohormones, only the jasmonic acid (JA) level increased in the SWT (but not *cork1*) roots (although not significantly), while the abscisic acid level increased in the *cork1* (but not SWT) roots upon treatment with 10 mM CB (Figure 11A,B). The highest ABA content was found in the *cork1* roots treated with 100 mM CB. The levels of all other phytohormones or their precursors were comparable in all samples.

Overall, these results indicate that the CB/CORK1 pathway promotes the accumulation of three amino acids (glutamic acid, glutamine, and asparagine), glucose, and ABA, while JA is downregulated (cf. Section 3). Higher JA levels in the SWT may indicate defense gene activation upon CB treatment, whereas stimulation of the ABA level by CB in the mutant suggests that it is stressed after cellooligomer application. The lower glucosinolate level in unchallenged *cork1* mutants suggests that CORK1 might have a function that does not require receptor activation.

## 3. Discussion 

### 3.1. CORK1 Localization and Inhibition of Cellulose Biosynthesis 

We demonstrated that *CORK1* is preferentially expressed in the vascular tissue of the upper part of the roots (Figure 1), i.e., tissue with differentiated cells and established cell walls. Expression in root tissue points to a specific role of this receptor kinase in sensing signals from the rhizosphere. It appears unlikely that the receptor kinase is part of the general cell wall integrity surveying system operating in the whole plant. Expression in the vascular root tissue might explain why the inhibition of cellulose biosynthesis by ISX did not stimulate *CORK1* expression (Figure 2), since de novo synthesis of cell wall material in the differentiated cell is low compared to the growing cells. Moreover, CORK1 is not only involved in cellooligomer perception but may also function as a coreceptor for the perception of mixed-linked β-1,3/1,4-glucan oligosaccharides [6]. The expression of *At1g56130* (*IGP2/3*) and *At1g56140* (*IGP4*), whose gene products are involved in the perception of mixed-linkage β-1,3/1,4-glucan oligosaccharides [6], is also not stimulated after the application of ISX (Figure 2). The three LRR-malectin receptor kinases might perceive cellooligomers or mixed-linked glucan oligosaccharides in the apoplast which accumulate when more damage- or stress-exposed peripheral cells of the roots are damaged, e.g., after a pathogen attack or due to stressful conditions in the rhizosphere. Cellooligomers in the root tissue might also derive from root-colonizing microbes, as shown in the case of *Piriformospora indica* [14], or decomposing material in the root environment. In the vascular tissue, CORK1 is protected against biotic and abiotic stresses by the epidermal and subepidermal cell layers. Therefore, this receptor kinase probably does not belong to the first line of defense against stress in Arabidopsis roots. 

Interestingly, the *At1g56120* message was upregulated 6 h after ISX application in the shoots (Figure 2), but not in the roots, indicating that the function of this receptor kinase might differ from *CORK1*, *At1g56130* (*IGP2*/*3*), and *At1g56140* (*IGP4*). The *At1g56120* expression profile is also different when compared to the expression profiles of the other three receptor kinase genes (www.arabidopsis.org, accessed on 15 November 2023). Furthermore, CORK1, At1g56130 (IGP2/3), and At1g56140 (IGP4) were found in mutant screens for the perception of cellooligomers or mixed-linked glucan oligosaccharides [5,6], while At1g56120 was not. The function of At1g56120, which is closely related to CORK1, IGP2/3, and IGP4, and is also a member of the LRR-malectin domain-receptor kinase subfamily [6,10], has to be elucidated.

### 3.2. Activation of CORK1 by Different Disaccharides

Non-plant malectins bind the disaccharides maltose and nigerose, which raises the question whether CORK1 activation is restricted to cellooligomers with β-1,4-bound glucose moieties, or whether other sugars can also induce cellular response in a CORK1-dependent manner. We found that—besides CT—only the disaccharides CB, and, to a much lesser extent, lactose (with a β-1,4 bond) and trehalose, can activate cellular processes in a CORK1-dependent manner (Figure 3, Figure 4 and Figure 5). The concentration of CB (1 mM) which is required for a detectable difference in the readouts between the SWT and *cork1* roots is at least 100 times higher than that for CT (10 µM), and those for lactose and trehalose (25 mM) are physiologically irrelevant. However, these results might give us hints for future biochemical studies of cellooligomer recognition by CORK1. Moreover, disaccharides are easily taken up by the cells and used as an energy source (cf. below). Therefore, understanding which disaccharide can induce cellular responses in a CORK1-dependent manner, besides being used as an energy source, is important to understand potential crosstalk with the primary sugar metabolism. Interestingly, maltose with an α-1,4 bond is active in mammals, while CT, CB, and lactose with β-1,4 bonds are active in plants. We assume that the ability to bind sugars with β-1,4 bonds is an evolutionary adaption of CORK1 to the plant cell wall chemistry, and that the high activity of the CT might ensure that mass disaccharides in the apoplast do not activate the receptor. During cellulose degradation, CB is an intermediate that is cleaved to two glucose moieties. The perception of CB would directly interfere with the primary sugar metabolism. CT is a nonabundant or very low-abundant intermediate during cellulose degradation by plant enzymes, but the abundance may differ when microbial enzymes degrade the plant cell wall. Thus, CT perception might inform the root cell about the presence of pathogenic microbes. Furthermore, at least one disaccharide without a 1–4 bond is recognized by non-plant malectin proteins (nigerose) and active in plants (trehalose).

### 3.3. Cellooligomer/CORK1 Signaling Exhibits Crosstalk with Chitin Signaling, FER, and BAK1

**Figure 12:**
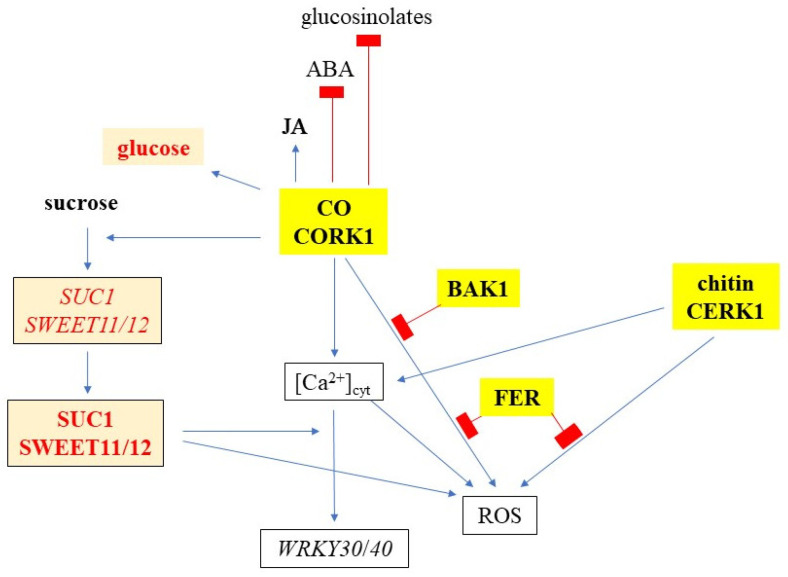
A model describing cellooligomer (CO)/CORK1-controlled responses, and the crosstalk with other receptor kinases (yellow boxes) and sugar metabolism/transport (orange boxes). Readouts include [Ca^2+^]_cyt_ elevation, ROS production, and *WRKY30/40* expression, while glucose, JA, ABA, and glucosinolate levels were assayed by metabolomics.

Figure 3 and Figure 4 suggest that CORK1 and CYCAM inhibits chitin-induced [Ca^2+^]_cyt_ elevation, while chitin-induced ROS production is inhibited by CYCAM, but not CORK1. No significant differences were observed for chitin-induced *WRKY30* and *WRKY40* expression between the wild-type and mutant seedlings (Figure 5). This indicates crosstalk between cellooligomer and chitin signaling which probably occurs at different levels. Yeh et al. [54] have shown that the Arabidopsis receptor-like kinase IMPAIRED OOMYCETE SUSCEPTIBILITY1 (IOS1), which also contains a malectin domain, physically associates with CERK1, and that IOS1 is critical for chitin-mediated PAMP-triggered immunity. Similarly, the malectin-like domain-containing receptor-like kinase FER promotes chitin signaling, whereas the RAPID ALKALINIZATION FACTOR23 (RALF23) ligand-bound FER plays an opposite role ([54], see below). Our physiological studies demonstrate that CORK1 is another LRR-malectin receptor kinase which interferes with chitin signaling, whereas chitin-induced [Ca^2+^]_cyt_ elevation (Figure 3) but not *WRKY30* expression was inhibited by CORK1 (Figure 5 and Figure 12). 

FER restricted ROS production induced by CT or chitin in roots (Figure 6). FER is involved in numerous physiological processes and regulated by a myriad of apoplastic and cytoplasmic factors [55,56,57,58,59]. The receptor kinase also modulates cellulose and starch content [60,61,62,63], and acts as a scaffold to promote PAMP-triggered immunity [64]; however, we did not observe that stimulation of *WRKY30*/*40* expression by chitin or CT was affected by FER (Supplementary Figure S3). FER is a receptor for RALF peptide ligands, in particular RALF1 and RALF23. Upon FER activation, a rapid increase in [Ca^2+^]_cyt_ elevation precedes NADPH oxidase-dependent ROS production [55,59]. The regulation of the NADPH oxidase by FER requires a GPI-anchored membrane-protein produced by LORELEI or LORELEI-like proteins [65]. The restriction of chitin-induced ROS production by FER might occur at extra- and/or intracellular levels. An example of an extracellular inhibition of the FER function provides the SITE1-PROTEASE which cleaves RALF propeptides. Without cleavage, FER facilitates the ligand-induced complex formation of the immune receptor kinases EF-TU RECEPTOR and FLS2 with their coreceptor BAK1 to initiate immune signaling [64]. Furthermore, IOS1 associates with BAK1 in a ligand-independent manner, and stimulates the FLS2-BAK1 complex formation upon PAMP treatment [54]. More recently, Gronnier et al. [66] have shown that FER regulates the plasma membrane nanoscale organization of FLS2 and BAK1. Akin to FER, extensin proteins contribute to RALF23 responsiveness and regulate BAK1 nanoscale organization and immune signaling. RALF23 perception leads to the rapid modification of FLS2 and BAK1 nanoscale organization, and its inhibitory activity on immune signaling relies on FER kinase activity. This example provides additional evidence for the flexibility of the PRR signaling network. Our data suggest that CT/CORK1 signaling is also part of this regulatory circuit. Protein interaction studies and the role of RALF peptide ligands are required to understand (i) the role of the extracellular environment for CORK1 function in the PRR network, (ii) which interactions occur at the plasma membrane, and (iii) which occur during D/PAMP-induced ROS production.

Besides FER, CT application also results in the rapid phosphorylation of BAK1 in a CORK1-dependent manner [5]. Furthermore, Zarrattini et al. [67] have shown that CB upregulates *BAK1*, *FER*, and *CERK1* to trigger innate immunity. Figure 6A,C demonstrates that CT-induced [Ca^2+^]_cyt_ elevation was not affected by *bak1* inactivation, while CT-induced ROS production was significantly lower in the *bak1* mutant than in the wild-type seedling. BAK1 might inhibit CT-activated NADH oxidase activity or the activities of other ROS-producing enzymes. Using gene silencing in *Nicotiana benthamiana*, Segonzaz et al. [68] have shown that the PAMP-triggered Ca^2+^ burst is upstream of separate signaling branches, one leading to MAPK activation and then gene expression, and another to ROS production. Likewise, CT-triggered ROS production may include post-translational modification operating at the NADH oxidase. The N-terminus of the NADH oxidase contains Ca^2+^ binding EF-hands, and the Ca^2+^ might be provided by CT- or chitin-triggered [Ca^2+^]_cyt_ elevation. In addition, NADPH oxidase activity is also regulated by phosphorylation, cysteine oxidation, S-nitrosation, phosphatidic acid, and ubiquitination [69,70]. Nitric oxide initiates a negative feedback loop limiting ROS production by NADH oxidases [71]. More research including the generation of double knock-out lines is required to understand how BAK1 specifically inhibits CT-induced ROS production and whether CT/CORK1 signaling affects NADH oxidase activity or the activities of other ROS-producing enzymes independently of [Ca^2+^]_cyt_ elevation.

### 3.4. CORK1 Restricts Sucrose-Induced Sugar Transporter Gene Expression

*SUC1* is highly expressed in roots, and its expression profile under different conditions suggests that the protein is involved in the uptake of sucrose, unloaded from the phloem, into growing root cells with no symplastic connection [72,73]. The stimulation of *SUC1* expression by sucrose is CORK1-dependent, and the inactivation of *CORK1* represses *SUC1* expression (Figure 7). These effects are even stronger for the *SWEET11* and *SWEET12* genes. SWEET11 and SWEET12 transport predominantly hexoses or sucrose, and pathogens induce *SWEET* genes to promote secretion of sucrose into the cell wall space, where it is used as an energy source after cleavage by cell wall invertases [35]. The downregulation of sucrose-induced *SWEET* gene expression in the *cork1* mutant lowers the apoplastic sugar level. Since less sugar is in the apoplast, SUC1 is less required for sucrose translocation from the apoplast into the root cells. Higher cellular sugar levels might promote the primary sugar metabolism in the cell. Although this requires further investigation, it is obvious that CORK1 interferes with the sugar (sucrose) transport in Arabidopsis roots (Figure 12).

Crosstalk between sugar transport and innate immunity or stress responses is well documented [13,35]. SWEET and STP transporters enhance or restrict disease through controlling the level of nutrients provided to pathogens [35]. ERD6 is a putative sucrose transporter and its gene is induced by dehydration and cold (TAIR, 2 January 2024). STPs are hexose-specific H^+^-symporters and involved in stress responses. STP4 is induced by wounding, STP13 is involved in programmed cell death, and STP13 resorbs hexoses to support the host with energy for defense compounds and to deprive apoplastic microbes by changing sugar fluxes toward host cells [38]. Furthermore, the transcriptional activation of *STR13* synchronizes biotic and abiotic stress signaling [37] and confers a powdery mildew resistance in *Medicago truncatula* [39]. SWEET11b protects rice plants against *Xanthomonas oryzae* [74], and cabbage SWEETs participate in chilling and clubroot disease responses [75]. Therefore, it is conceivable that cellulose degradation products activate both transport and immune responses, and that crosstalk between these signaling process ensures synchronized responses. Furthermore, sugar metabolites trigger immune responses. CB elicits immunity in lettuce [46] and Arabidopsis [51], and stimulates *SUC1* and *STP4* expression [67]. Fructans prime ROS dynamics and *Botrytis cinerea* resistance [76], while trehalose modulates defense responses in Arabidopsis [40,42]. Our data show that CT/CORK1 alters the expression or phosphorylation of sugar transporter genes and thus the sugar distribution, and mutations in sugar transporter genes impact CT/CORK1-induced responses in Arabidopsis roots (Figure 7, Figure 8 and Figure 12; Supplementary Tables S5 and S6). Furthermore, the levels of some sugar metabolites (Figure 9), as well as the phosphorylation state of enzymes involved in the primary sugar metabolism, respond to cellooligomer applications to the roots of wild-type seedlings but not *cork1* mutant seedlings (Supplementary Table S7). 

### 3.5. Cellooligomer/CORK1 Signaling Alters the Amino Acid, Sugar, Glucosinolate, and Phytohormone Patterns in Arabidopsis Roots

The glutamic acid, glutamine, and asparagine levels are slightly upregulated after CORK1 activation (Figure 9). These amino acids play crucial roles in nitrogen metabolism, which is intricately linked to stress and defense responses in plants [77]. Glutamine, the first amino acid synthesized in nitrogen assimilation in plants, is the building block for protein synthesis and an N-donor for the biosynthesis of amino acids, nucleic acids, amino sugars, vitamin B coenzymes, and N-containing secondary metabolites [78]. The amino acid induces immune responses in Arabidopsis, by stimulating the expression of wound-, defense-, and stress-related genes [79]. Moreover, the application of glutamate to roots activates PAMP-, salicylic acid-, and JA-inducible genes and primes chitin-induced responses in leaves, possibly through the transcriptional activation of the chitin receptor *LYK5* [79]. Asparagine belongs to the high-abundant amino acids which are synthesized during abiotic stress to act as osmolytes [80]. The asparagine synthetase 2 mutant exhibits low salt stress tolerance [81] and the amino acid is imbedded into a signaling network involved in stress resistance in Arabidopsis [82,83]. Glucose is an important regulator of plant defense, e.g., by controlling the interaction and phosphorylation of BRI1 and BAK1 [84]. Also, the phytohormone ABA is a well-investigated abiotic stress hormone [85]. In our results, although the [Ca^2+^]_cyt_ level is not elevated, the ABA level is stimulated in the CB-exposed *cork1* roots (Figure 11). Either both responses are independently regulated by the cellooligomer/CORK1 pathway, or the [Ca^2+^]_cyt_ pool induced by this pathway is different from the pool required for many ABA responses. Elevated ABA levels in the *cork1* roots upon 100 mM CB application indicates that the mutant suffers under stress. Since ABA does not increase if SWT roots are exposed to 100 mM CB, the activated CORK1 might diminish the stress situation.

Souza et al. [51] have demonstrated that CB-treated roots exhibit the upregulation of *LIPOXYGENASE* (*LOX)3* and *LOX4*, genes encoding proteins in the octadecanoid pathway leading to the production of JA. This finding aligns with the observed increase of the JA level in the wild-type, but not the *cork1* roots (Figure 11). Interestingly, the levels of the JA precursor *cis*-OPDA and the physiologically active JA-isoleucine did not respond to the cellooligomer/CORK1 pathway. Either they are not the primary targets of CB-induced signaling, or the elevated physiologically inactive JA level is established to prime stress response in local and/or systemic tissue.

The lower glucosinolate levels in CB-exposed wild-type roots may indicate a trade-off between stress and growth responses. Glucosinolates are mainly involved in defense responses, while cellooligomers are DAMPs which respond to cell wall damage. Therefore, cellooligomer-exposed roots might prioritize the allocation of resources for growth and/or cell wall repair rather than defense compounds. The lower glucosinolate levels in mutant roots without exposure to cellooligomers are not clear. A possible explanation could be that CORK1 is a positive regulator of glucosinolate biosynthesis [86], and the binding of the cellooligomer to CORK1 or inactivation of the genes represses CORK1 function. Overall, the majority of the data are consistent with the idea that the cellooligomer/CORK1 pathway targets those metabolite levels which counteract stress.

## 4. Materials and Methods

### 4.1. Plant Material and Growth Conditions

The Arabidopsis *cork1*-2 insertion mutant line (N674063; SALK_021490C) was obtained from Nottingham Arabidopsis Stock Center (NASC). Homozygous seedlings were crossed with the aequorin-containing wild-type line pMAQ2. The corresponding SWT and homozygous seedlings from the F3 generation were used for experiments, as earlier described in [5]. The seeds of these lines were surface-sterilized for 8 min in sterilizing solution containing lauryl sarcosine (1%) and Clorox cleaner (23%). The surface-sterilized seeds were washed with sterilized water eight times and placed on Petri dishes with a Murashige and Skoog medium (Duchefa Biochemie, Haarlem, The Netherlands) supplemented with 0.3% gelrite [87]. After cold treatment at 4 °C for 48–72 h, the plates were incubated at 22 °C under long day conditions (16 h light/8 h dark; 80 µmol m^−2^ s^−1^). In addition, insertion lines in the genes for SUC1, SWEET11, SWEET12 and the double insertion line in the genes for SWEET11 and SWEET12 were used in this study [72]. The cytoplasmic Ca^2+^ elevation mutant (*cycam*), as well as the aequorin-containing *bak1* and *cerk1* insertion lines were generated or described in an earlier study [14,88]. 

*Arabidopsis thaliana* ecotype Col-0 was used as a control for ISX treatment and for expression analysis in *feronia* (*fer*) mutants. The *fer2* [89] and *fer4* (N69044) mutants were provided by Dr. Judith Fliegmann (ZMBP, University of Tübingen). Twelve-day-old seedlings were transferred to PNM with a nylon membrane. The next day, the plants were mock treated, or treated with 0.6 μM ISX by pipetting the solution to the roots. Both treatments had an equal amount of dimethyl sulphoxide (DMSO). The roots and shoots were harvested separately 6 and 9 h after the treatment.

### 4.2. Chemicals for Elicitation

CT (C1167, Sigma-Aldrich, St. Louis, MO, USA, or 0-CTR-50MG, Megazyme, Wicklow, Ireland) and chitin (chitohexaose, OH07433 [Carbosynth]) were dissolved in distilled water to make a 10 µM working solution. CB (528-50-7, Carl Roth, Germany) dissolved in distilled water was used at a concentration of 1 mM. Other sugars (sucrose, glucose, galactose, trehalose, maltose, fructose) were also purchased from Carl Roth (Karlsruhe, Germany) while lactose was obtained from Merck (Darmstadt, Germany). These sugars were used at a concentration of 25 mM. ISX (36138, Sigma-Aldrich) was dissolved in DMSO to make a stock of 5 mM and used at a final concentration of 0.6 µM. PEG 6000 was purchased from Carl Roth (Karlsruhe, Germany).

### 4.3. ROS Measurements

ROS was detected using a luminol-based chemiluminescence assay as described previously [5]. Briefly, the seedlings were grown vertically for 14 days on a Hoagland medium with agar (Hoagland’s No. 2 Basal Salt Mixture; Sigma-Aldrich). The root tissues were incubated in 150 µL of autoclaved double distilled water in a 96-well plate in the dark. After 90 min, the water was gently replaced with 150 µL of working solution containing 2 µg/mL horseradish peroxidase (Sigma-Aldrich) and 100 µM luminol derivative, L-012 (FUJIFILM Wako Chemicals Europe GmbH, Neuss, Germany). Luminescence from each well was measured over 40 min after the addition of elicitors using a luminometer (Luminoskan Ascent v2.4, Thermo Fisher Scientific, Dreieich, Germany), and the results are displayed as relative light units (RLUs). Eight plants were used per treatment and three independent replicates were performed.

### 4.4. Cytosolic Calcium Measurements

The seedlings were grown vertically on a Hoagland medium and an entire, individual root from 15-day-old seedlings expressing aequorin was incubated overnight in 150 µL of 7.5 μM coelenterazine solution (P.J.K. GmbH, Kleinblittersdorf, Germany) in the dark for reconstitution [90,91]. The luminescence counts were recorded using a microplate luminometer (Luminoskan Ascent v2.4, Thermo Fisher Scientific, Dreieich, Germany). After the measurement, a discharging solution containing 1 M CaCl_2_ and 10% ethanol (*v*/*v*) was added to estimate the amount of residual aequorin. Cytosolic calcium concentrations were calculated using the equation of Rentel and Knight [92].

### 4.5. RNA Isolation, cDNA Synthesis, and RT-qPCR

10-day-old (n = 20–24) excised root samples were treated with the above-mentioned concentrations of sugars for one hour. The roots were harvested and homogenized in liquid nitrogen. RNA was extracted with TrizolTM (Thermo Fisher Scientific, Waltham, USA) and chloroform according to the manufacturer’s protocol. The RNA isolation was followed by an additional cleaning step to remove excess salts using 3 M sodium acetate (1/10 (*v*/*v*) in RNase-free water, pH = 5.2), 600 μL of ice-cold ethanol, and overnight incubation at −20 °C. One µg of total RNA was reverse-transcribed with a RevertAid RT Reverse Transcription Kit (Thermo Fisher Scientific).

Dream Taq DNA Polymerase (Thermo Fisher Scientific, Germany) and Evagreen^®^ (Biotium, Fremont, CA, USA) were used for quantitative reverse transcription PCRs (RT-qPCRs). CFX ConnectTM Real-Time PCR Detection System (Bio-Rad, Feldkirchen, Germany) was used for running and analyzing qPCRs. The expression of genes was normalized to the housekeeping gene encoding a ribosomal protein (RPS; At1g34030), using the 2^−∆∆CT^ method [93]. All primers used are mentioned in Supplementary Table S3.

### 4.6. Generation of the CORK1 Promoter::GFP Fusion Construct and Plant Transformation

The plant tissue was homogenized and DNA extraction was performed according to [94]. An approximately 1.5 kb promoter region of *CORK1* (At1g56145.1) (starting one nucleotide before the ATG codon) was amplified from *Arabidopsis thaliana* genomic DNA by PCR using Phusion^TM^ High-Fidelity DNA Polymerase (Thermo Fisher Scientific) with *SacI* and *SpeI* restriction sites in the forward and reverse primers, respectively. The promoter fragment was digested using *Sac1* and *Spe1* restriction enyzmes. The *GFP* fragment was amplified using EGFP_Spe1_FWD, T35S_Xma1_REV, and digested using *Spe1* and *Xma1*. pB7FWG2 was digested with *SpeI* and *XmaI* and ligated with the PCR fragment SpeI-EGFP-T35S-XmaI. Then, this plasmid was digested with *SacI* and *SpeI*, and ligated with the PCR fragment SacI-pCORK1-SpeI to create the pCORK1::EGFP construct. The primers used are listed in the Supplementary Table S3. The promoter sequence was confirmed by Sanger sequencing (Eurofins Genomics, GmBH, 85560 Ebersberg, Germany). The transformation of Arabidopsis was performed by floral dip with the Agrobacterium GV3101 strain as described previously [95]. Transgenic plants were selected using BASTA (Bayer AG, Leverkusen, Germany).

### 4.7. Confocal Microscopy

The fourteen-day-old Arabidopsis seedlings were examined using an LSM 880 microscope (Zeiss Microscopy GmbH, München, Germany) with the 488 nm laser line produced by an argon multiline laser (11.5 mW). The images were taken by use of a 40× objective (Plan-Apochromat 40_/0.8). Lambda stacks were created using the 32 channel GaAsP detector followed by linear unmixing with the ZEN (black) 2.3 SP1 software. Z-stacks were taken from relevant areas of the samples and maximum intensity projections were produced with the ZEN software (Zeiss Microscopy GmbH).

### 4.8. Long Term Exposure to CB and CT

The SWT and *cork1* mutant seedlings were grown on half Murashige and Skoog media for 9 days, and on the 10th day, they were transferred to PNM either without CB/CT (control) or with filter-sterilized CB (1 mM, 10 mM, 30 mM, 50 mM, 100 mM) and grown for an additional 6 days. For the CT experiment, 10 µM of filter-sterilized CT was added to PNM media. The roots of 15-day-old plants from both experiments (10 mM CB or 10 µM CT) were harvested separately, and weights were measured and used for phytohormone, sugars, and glucosinolate analyses.

### 4.9. Short Term Exposure to CB 

The SWT and *cork1* mutant seedlings were grown for 14 days on half Murashige and Skoog media. An amount of 48–60 seedlings were then transferred to PNM media (control), PNM with PEG 6000, CB (10 mM), or CB (100 mM). An amount of 15 mM PEG 6000 was used, as its osmotic potential is the same as that of 100 mM CB. PEG 6000 was used to eliminate the effect of osmotic stress shock on downstream responses caused by high amounts of CB. The plants were harvested after 6 h and used for sugar, amino acid, glucosinolate, and phytohormone analyses. 

### 4.10. Phytohormone Analyses by Lipid Chromatography (LC)-Mass Spectrometry (MS)/MS 

To quantify phytohormone profiles, 150–210 mg of fresh frozen tissue was extracted with 1 mL of 80% methanol (*v*/*v*) containing 40 ng D4-SA (Santa Cruz Biotechnology, Dallas, TX, USA), 40 ng D6-ABA (Toronto Research Chemicals, Toronto, ON, Canada), 40 ng D6-JA, 8 ng D6-JA-Ile (both HPC Standards GmbH, Cunnersdorf, Germany) and 40 ng of D5-indole-3-acetic-acid (OlChemIm s.r.o., Olomouc, Czech Republic). Phytohormone analysis was performed on an Agilent 1260 series high-performance LC (HPLC) system (Agilent Technologies, Santa Clara, CA, USA) as in [96], with the modification that a tandem mass spectrometer QTRAP 6500 (SCIEX, Darmstadt, Germany) was used in multiple reaction monitoring (MRM) mode with parameters listed in Supplementary Table S4. Concentrations of *cis*-(+)-12-oxo-phytodienoic acid (*cis*-OPDA) and OH-JA were determined relative to the quantity of the internal standard D6-JA applying a response factor (RF) of 1.0, while OH-JA-Ile and COOH-JA-Ile were quantified relative to D6-JA-Ile, applying an RF of 1.0. Since we observed that both the D6-labeled JA and D6-labeled JA-Ile standards (HPC Standards GmbH, Cunnersdorf, Germany) contained 40% of the corresponding D5-labeled compounds, the sum of the peak areas of the D5- and D6-labeled compounds was used for quantification. Indole-3-acetic acid (IAA) was measured with the same LC-MS system and the same chromatographic parameters, but ionization was in positive mode with MRM parameters, as shown in Supplementary Table S5.

### 4.11. Sugar Measurements 

Soluble sugars were analyzed from the 80% (*v*/*v*) methanol extracts for phytohormone analysis at 1:10 dilution in water containing 5 µg/mL ^13^C_6_-glucose (Sigma-Aldrich) and 5 µg/mL ^13^C_6_-fructose (Toronto Research Chemicals, Toronto, Canada), by LC-MS/MS as described in [97]. Sugars were analyzed using an Agilent 1200 HPLC system equipped with an API3200 tandem mass spectrometer (AB Sciex, Darmstadt, Germany). The HPLC was equipped with a hydrophilic interaction liquid chromatography (HILIC) column (apHera-NH2 Polymer; Supelco, Bellefonte, PA, USA), and chromatographic separation was performed using water and acetonitrile as mobile phases A and B, respectively, with a flow rate of 1.0 mL min^−1^. The column temperature was maintained at 20 °C. The mass spectrometer equipped with a turbo spray ion source was operated in the negative ionization mode. The ion spray voltage was maintained at −4500 eV and the turbo gas temperature was set at 600 °C. Nebulizing gas was set at 60 psi, curtain gas at 40 psi, heating gas at 60 psi, and collision gas at a medium level. Multiple reaction monitoring (MRM) was used to monitor the analyte parent ion to product ion formation (Supplementary Table S6). Data were acquired using the software Analyst 1.5.1 and quantification was performed using the software MultiQuant 3.0.3 (Sciex, Framingham, MA, USA). The concentrations of glucose and fructose were determined relative to the internal standards of ^13^C_6_-glucose and ^13^C_6_-fructose, respectively. The contents of sucrose, trehalose (both from Sigma-Aldrich), and mannitol (Honeywell Fluka, Marxen, Germany) were calculated based on external standard curves.

### 4.12. Quantifcation of Glucosinolates

Glucosinolate concentrations were quantified using the same raw extracts as used to quantify phytohormone profiles, and analyzed by HPLC-ultraviolet detection (UV) as described in [98]. In short, a 700 µL aliquot of the 80% methanol raw extract for phytohormone analysis was loaded onto DEAE Sephadex A-25 columns and treated with arylsulfatase for desulfation (Sigma-Aldrich) [99]. The eluted desulfoglucosinolates were separated using HPLC (Agilent 1100 HPLC system, Agilent Technologies) on a reversed phase C-18 column (Nucleodur Sphinx RP, 250 × 4.6 mm, 5 µm, Machrey-Nagel, Düren, Germany) with a water (A)–acetonitrile (B) gradient (0–1 min, 1.5% B; 1–6 min, 1.5–5% B; 6–8 min, 5–7% B; 8–18 min, 7–21% B; 18–23 min, 21–29% B; 23–23.1 min, 29–100% B; 23.1–24 min 100% B, and 24.1–28 min 1.5% B; flow 1.0 mL min^−1^). Detection was performed with a photodiode array detector and peaks were integrated at 229 nm. Desulfated glucosinolates were identified by comparison of the retention time and UV spectra to those of purified standards previously extracted from *Arabidopsis thaliana* [99]. We used the following molar response factors for the quantification of individual glucosinolates relative to the internal standard (4-hydroxybenzyl glucosinolate): aliphatic glucosinolates 2.0, indole glucosinolates 0.5 [93].

### 4.13. Amino acid Analysis 

Amino acids were quantified with an LC-MS/MS using a C18-column (XDB-C18, 50 × 4.6 mm × 1.8 µm; Agilent, Santa Clara, CA, USA) after diluting the 80% methanol extracts from phytohormone analysis by 1:10 (*v*:*v*) with water containing 10 µg ml^−1^ of a mixture of ^15^N/^13^C labeled amino acids (Isotec, Miamisburg, OH, USA) and 5 µM of D5-tryptophane (Cambridge Isotope Laboratories, Inc., Andover, MA, USA). For details on the chromatography and mass spectrometry (Agilent 1260 LC system; Agilent Technologies, Santa Clara, CA, USA) coupled with a QTRAP 6500 tandem mass spectrometer (SCIEX, Darmstadt, Germany), see [100] and Supplementary Table S7. The mass spectrometer was operated in positive ionization mode in multiple reaction monitoring mode. All amino acids were quantified relative to the peak area of the corresponding isotopically-labeled compound, except for asparagine (using isotopically-labeled aspartate and a response factor of 1.0).

### 4.14. Statistical Analysis

GraphPad PRISM 9 and ORIGINPRO 2023 softwares were used for data analysis and plotting graphs. The statistical details of the experiments are specified in the figure legends. Statistical significance of data under normal distribution was tested using a two-tailed unpaired Student’s *t*-test for pairwise comparisons. For experiments involving one or two classes of factors, statistically significant differences were calculated using one- and two-way analysis of variance with the Holm–Sidak multiple comparison test and Tukey’s post hoc test. Normality was verified using the Shapiro–Wilk test. Data that did not pass this test were subjected to log transformation or the Kruska–Walis test. The significance threshold was set at *p* < 0.05. Figures were arranged with LibreOffice Draw 5.1.6.2.

## 5. Conclusions

We present physiological evidence that the cellooligomer/CORK1 signaling pathway exhibits crosstalk with PAMP- and DAMP-triggered immune responses and the sugar metabolism in Arabidopsis roots. Further studies are required to confirm this at the molecular and genetic levels. 

## Data Availability

Raw sequences for the GWAS have been deposited to the NCBI Gene Expression Omnibus (GEO) database (accession no. GSE197891). For transcriptome analysis, raw sequences and the count tables after DESeq2 analysis have been deposited to the Gene Expression Omnibus (GEO) database (accession no. GSE198092). The mass spectrometry proteomics data have been deposited to the ProteomeXchange Consortium via the PRIDE partner repository [101] with dataset identifier PXD033224.

## Supplementary Materials

The following supporting information can be downloaded at: https://www.mdpi.com/article/10.3390/ijms25063472/s1.
