# Supplementary material for: Cellooligomer/CELLOOLIGOMER RECEPTOR KINASE1 Signaling Exhibits Crosstalk with PAMP-Triggered Immune Responses and Sugar Metabolism in Arabidopsis Roots
Source: Int J Mol Sci. 2024 Mar 19;25(6):3472. doi: 10.3390/ijms25063472 (PMC10970632; doi:10.3390/ijms25063472)
Supplement: Supplementary file 1 [file ijms-25-03472-s001.zip › ijms-2910705-supplementary.pdf]

Supplementary Materials

Supplementary Table S1: Sugar-related genes which are regulated by CT in Arabidopsis wild-type roots but not in the *cork1* mutant.
Supplementary Table S2: Phosphoproteins involved in sugar metabolism which are regulated by CT in SWT, but not in *cork1* roots.
Supplementary Table S3: Primers.
Supplementary Table S4: Details of the analysis of phytohormones by LC-MS/MS using an Agilent HPLC 1260/QTRAP6500 (Sciex) instrument in negative ionisation mode.
Supplementary Table S5: Details of the analysis of Indole-3-acetic acid by LC-MS/MS in positive ionization mode.
Supplementary Table S6: Details of analysis of sugars by LC-MS/MS in negative ionization mode.
Supplementary Table S7: Details of analysis of amino acids by LC-MS/MS in positive ionization mode.
Supplementary Figure S1: Root fresh weights, sugar, glucosinolate and phytohormone levels of wild-type and *cork1* seedlings grown on different CB concentrations.
Supplementary Figure S2: Root fresh weights, sugar, glucosinolate and phytohormone levels of wild-type and *cork1* seedlings exposed to medium with 10 µM CT for 6 days.
Supplementary Figure S3: Upregulation of (C) *WRKY30* and (D) *WRKY40* mRNA levels in root tissue 1 h after treatment with 10 µM CT or 10 µM chitin in *fer-2* and *fer-4* mutants.

Supplementary Table S1. Sugar-related genes which are significantly (p<0,05) up- (yellow) or down- (blue) regulated in Arabidopsis wild-type roots 1 h after application of 10 µM CT in comparison to the water control, but not in the *cork1* mutant. Based on 3 independent experiments [1] (-), no regulation. The raw sequences are available at the NCBI Gene Expression Omnibus (GEO) database (accession no. GSE197891).

No	Accession No	Sugar transporter	Log2 fold in SWT	Log2 fold in *cork1*
1	At4g36670	Polyol/monosaccharide transporter 6 (PMT6/PLT6)	1.88	0.14
2	At1g08930	Early Response to Dehydration Six (ERD6)	1.85	0.21
3	At5g26340	Sugar transport protein 13 (STP13)	1.14	-0.08
4	At1g71880	Sucrose-proton symporter 1 (SUC1)	1.07	-
5	At3g19930	Sugar transport protein 4 (STP4)	0.75	0.23
6	At5g40390	Probable galactinol-sucrose galactosyltransferase 5	0.63	-
7	At1g22710	Sucrose-proton symporter 2 (SUC2)	0.54	-
8	At1g08920	ERD (early response to dehydration) six-like 1	0.44	-0.16
9	At1g11260	Sugar transport protein 1 (STP1)	0.55	-
10	At3g52340	Sucrose-6-phosphate phosphohydrolase 2	0.53	-
11	At2g02860	Sucrose transporter 2 (SUT2)	0.41	-

Supplementary Table S2. Proteins involved in sugar metabolism with significant alteration in phosphorylation upon 10 µM CT treatment compared to water control in SWT, but not in *cork1* roots. Based on 3 independent experiments [1,2]. The mass spectrometry proteomics data are available at the ProteomeXchange Consortium via the PRIDE partner repository with dataset identifier PXD033224.

UniProt Accession No.	Protein	Sequence	WT-CT/ WT-H_2O (5min)	WT-CT/ WT-H_2O (15min)	*cork1*-CT/*cork1*-H_2O (5min)	*cork1*-CT/*cork1*-H_2O (15min)	*cork1*-CT/ WT-CT (5min)	*cork1*-CT/ WT-CT (15min)
Q94BT0	Sucrose-phosphate synthase 1 (SPS1)	SSPSLLLR	3.1	2.410	-1.604	-1.265	-1.893	-1.683
Q9FX32	Sucrose synthase 6 (SUS6)	VVSGIDVFDPK	1.410	6.061	2.104	1.542	1.206	1.752
F4K470	Galactinol-sucrose galactosyltransferase (DIN10)	KESPIFR	7.180	10.663	-1.003	1.685	-2.003	-4.483
Q94BT0	Sucrose-phosphate synthase 1 (SPS1)	GDIISDISTHGESTKPR	3.323	14.159	-2.655	-1.045	-7.830	1.149
Q9FY54	Sucrose-phosphate synthase 2 (SPS2)	VEFENSDSDSPSDSLRDINDISLNLK	1.183	3.028	1.118	-1.134	1.649	-2.504
Q944A6	Sucrose nonfermenting 4-like protein (SNF4)	TADPSQEAVPRMSGVDLELSR	1.907	-138.288	41.933	2.219	3.060	168.826
Q94BT0	Sucrose-phosphate synthase 1 (SPS1)	INSAESMELWASQQK	39.805	1.279	-1.635	1.027	1.335	-1.303
Q96290	Monosaccharide-sensing protein 1 (MSSP1)	YYLKEDGAESR	7.070	-1.817	3.290	-1.506	4.473	2.095
O82587	Sugars will eventually be exported transporter 12 (SWEET12)	LGTLTSPEPVAITVVR	-4.265	2.358	1.313	-1.130	4.316	-2.872
O04249	Sugar transport protein 7 (STP7)	MAGGSFGPTGVAK	1.418	-1.059	-2.106	-1.492	-2.160	1.010
O04036	Early Response to Dehydration Six (ERD6)	LRGSDVDISR	2.485	-1.139	1.602	-1.082	1.410	2.720

Supplementary Table S3. Primers.

Primers used to generate the *CORK1* promoter::*GFP* fusion construct.

**Primer Name**	**Sequence**
Sac1_pAt1g56145_FWD	AAAAAGAGCTCTTCTTCCTTTCCTTCTCTGA
SpeI_pAt1g56145_REV	AAAAACTAGTCGTCGACGACCAAAGATGTGA
EGFP_Spe1_FWD	AAAAACTAGTTGATATCAATGGTGAGCAAGG
T35S_Xma1_REV	AAAAACCCGGGAGGTCACTGGATTTTGGTT
p*CORK1*_MF2	ACTAATGATTAACAAGTAAACAACGAC
GFP –seq REV	TGCAGATGAACTTCAGGGTCAG
Primers for gene expression analysis by quantitative PCR (qPCR)	
**Primer Name**	**Sequence**
RPS-qF<br>*RPS*-qR	GTCTCCAATGCCCTTGACAT<br>TCTTTCCTCTGCGACCAGTT
WRKY30-qF<br>*WRKY30*-qR	CGGAGCCAAATTTCCAAGAGG<br>GACGGAGAGTTTGATGCTGAG
WRKY40-qF<br>*WRKY40*-qR	AGCCCTCCCAAGAAACGCAAATC<br>GCTTGGAGCACAAGCACATTTGAAG
FRK1-qF<br>*FRK1*-qR	CGGTCAGATTTCAACAGTTGTC<br>AATAGCAGGTTGGCCTGTAATC
CYP81F2-qF<br>*CYP81F2*-qR	GTGAAAGCACTAGGCGAAGC<br>ATCCGTTCCAGCTAGCATCA
SUC1-qF<br>*SUC1*-qR	GACCTTTCGACGCCTTGTTC<br>AATACTCCACTAATCGCCGCTG
SWEET11-qF<br>*SWEET11*-qR	GCCAATCTCAGTGGTTCGTCAAG<br>GAAGAGGACTGCTTGCCATGT
SWEET12-qF<br>*SWEET12*-qR	CTCACATCTCCTGAACCAGTAGC<br>TGCAGCACTGTTTCTAACTCCC
At1g56120-qF<br>Atg56120-qR	AGACACTAGCTTCTCGACTG<br>ACAATCTTCATCTTCCCTCC
At1g56130-qF<br>Atg56130-qR	ACACAACAGGCTATTCCATGA<br>TCATCTTACATATATACAAACATCTC
At1g56140-qF<br>At1g56140-qR	ACAGGCTTCTGAATCCTTCAC<br>AGAATAAAGTGAACCTCATATACAC
At1g56145 (*CORK1*)-qF<br>At1g56145 (*CORK1*)-qR	ACCAGAGTACGTGATGCTTG<br>TCCATGCCCATTCGAGAAG

Supplementary Table S4. Details of the analysis of phytohormones by LC-MS/MS using an Agilent HPLC 1260/QTRAP6500 (Sciex) instrument in negative ionisation mode. Abbreviations are: Q1, selected *m/z* of the first quadrupole; Q3, selected *m/z* of the third quadrupole; RT, retention time; DP, declustering potential (V); and CE, collision energy (V); SA, salicylic acid; JA, jasmonic acid; ABA, abscisic acid; JA-Ile, jasmonic acid-isoleucin conjugate; OPDA, 12-oxo phytodienoic acid; OH-JA, 12-hydroxy-jasmonic acid; OH-JA-Ile, 11/12-hydroxy-jasmonic acid-isoleucin conjugate; COOH-JA-Ile, 11/12-carboxy-jasmonic acid-isoleucin conjugate; D4-SA, D4-salicylic acid; D6-JA, D6-jasmonic acid; D5-JA, D5-jasmonic acid; D6-ABA, D6-abscisic acid; D6-JA-Ile, D6-jasmonic acid-isoleucin conjugate; D5-JA-Ile, D5-jasmonic acid-isoleucin conjugate.

**Q1**	**Q3**	**RT (min)**	**Compound**	**Internal Standard**	**DP**	**CE**
136.93	93	3.3	SA	D4-SA	-20	-24
209.07	59	3.6	JA	D6-JA	-20	-24
263	153.2	3.4	ABA	D6-ABA	-20	-22
322.19	130.1	3.9	JA-Ile	D6-JA-Ile	-20	-30
290.9	165.1	4.6	OPDA	D6-JA	-20	-24
225.1	59	2.6	OH-JA	D6-JA	-20	-24
338.1	130.1	3.0	OH-JA-Ile	D6-JA-Ile	-20	-30
352.1	130.1	3.0	COOH-JA-Ile	D6-JA-Ile	-20	-30
140.93	97	3.3	D4-SA		-20	-24
215	59	3.6	D6-JA		-20	-24
214	59	3.6	D5-JA		-20	-24
269	159.2	3.4	D6-ABA		-20	-22
328.19	130.1	3.9	D6-JA-Ile		-20	-30
327.19	130.1	3.9	D5-JA-Ile		-20	-30

Supplementary Table S5. Details of the analysis of indole-3-acetic acid by LC-MS/MS. An Agilent HPLC 1260/QTRAP6500 (SCIEX) instrument was used in positive ionisation mode. Abbreviations are: Q1, selected *m/z* of the first quadrupole; Q3, selected *m/z* of the third quadrupole; DP, declustering potential (V); and CE, collision energy (V).

**Q1**	**Q3**	**Dwell Time (sec)**	**Compound**	**DP**	**CE**
176	130	20	IAA	40	19
181	135	20	D5-IAA-fragment135	40	19
181	134	20	D5-IAA-fragment134	40	19
181	133	20	D5-IAA-fragment133	40	19

Supplementary Table S6. Details of analysis of sugars by LC-MS/MS [HPLC 1200 (Agilent Technologies)-API3200 (AB SCIEX)] in negative ionization mode

Q1	Q3	RT (min)	Compound	DP	CE
178.8	89.0	6.7	Glucose	-25	-10
178.801	89.0	5.6	Fructose	-25	-12
340.9	59.0	8.2	Sucrose	-45	-46
340.9	59.0	8.9	Trehalose	-45	-46
503.1	179.0	10.5	Raffinose	-75	-28
181.0	89.0	6.3	Mannitol	-35	-22
185.0	92.0	6.7	13-C6-Glucose	-25	-10
185.01	92.0	5.6	13-C6-Fructose	-25	-12

Supplementary Table S7. Details of analysis of amino acids by LC-MS/MS [HPLC 1260 (Agilent Technologies)-QTRAP6500 (SCIEX)] in positive ionization mode

Compound	Q1	Q3	RT (min)	Internal Standard	IS Q1	IS Q3	DP	CE
Ala	90.1	44.1	0.5	13C,15N-Ala	94.1	47.1	20	17
Ser	106.0	60.1	0.5	13C,15N-Ser	110.0	63.1	20	15
Pro	116.1	70	0.5	13C,15N-Pro	122.1	75.0	20	19
Val	118.1	72.2	0.5	13C,15N-Val	124.1	77.2	20	13
Thr	120.1	74.2	0.5	13C,15N-Thr	125.1	78.2	20	13
Ile	132.2	86.1	1.1	13C,15N-Ile	139.2	92.1	20	13
Leu	132.2	86.1	1.3	13C,15N-Leu	139.2	92.1	20	13
Asp	134.1	74.1	0.5	13C,15N-Asp	139.1	77.1	20	19
Glu	148.1	102.1	0.5	13C,15N-Glu	154.1	107.1	20	15
Met	150.2	104.1	0.7	13C,15N-Met	156.2	109.1	20	13
His	156.2	110.1	0.4	13C,15N-His	165.2	118.1	20	17
Phe	166.2	120.2	2.6	13C,15N-Phe	176.2	129.2	20	17
Arg	175.1	70.1	0.4	13C,15N-Arg	185.1	75.1	20	31
Tyr	182.1	136.2	1.4	13C,15N-Tyr	192.1	145.2	20	17
Asn	133.1	74.1	0.5	13C,15N-Asp			20	21
Gln	147.1	130	0.5	13C,15N-Gln	154.1	136.0	20	13
Trp	205.2	188.1	3.2	D5-Trp	210.0	193.0	20	13

Supplementary Figures S1 and S2

When 9-day old SWT and *cork1* seedlings were exposed to 10 μM CT for 6 days, the shoot and root fresh weights of *cork1* was lower than those of the SWT, although the results were not significant (Figure S2, A and B). Moreover, CT application significantly inhibited root, but not shoot growth of SWT but not *cork1* seedlings (Figure 2). This is consistent with the observation that *CORK1* is preferentially expressed in roots (Figure 1). The low CT concentration (10 μM CT) had no effect on the primary sugar metabolism, since the sucrose, glucose, and fructose levels were not significantly different in SWT and *cork1* roots in the presence or absence of CT (Figure 2B). Also, the defense-related glucosinolate levels were not significantly different in the roots of CT-treated or -untreated SWT and *cork1* seedlings, although there was the tendancy that CT stimulated the glucosinolate levels in SWT, but not *cork1* roots (Figure 2C). Since longer exposure of the seedlings to 10 μM CT caused unspecific effects (in particular during germination and in very young seedlings), we tested whether the growth/stress response balance is altered by CB.

9-day old SWT and *cork1* seedlings were grown on different CB concentrations for 6 days. Compared to the water control, 10 mM CB stimulated root growth of SWT seedlings, while 30, 50 and 100 mM CB inhibited root growth in SWT seedlings No significant differences of the root fresh weights were observed for *cork1* seedlings exposed to different CB concentrations (Figure S1). Considering that at least 100-times more CB than CT is required to induce comparable responses in the roots (Figures 3 and 5A), an inhibitory effect of CB is only expected with concentrations which are much higher than 10 μM used for CT.

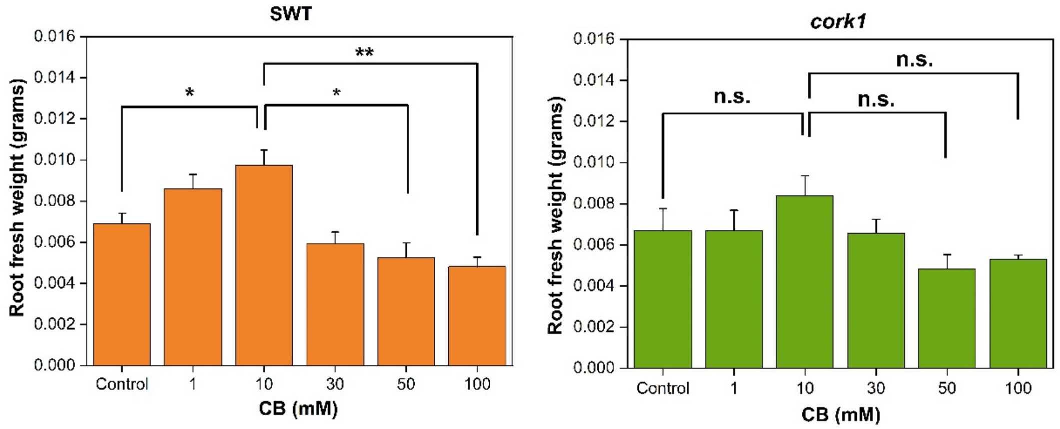

(**A**)

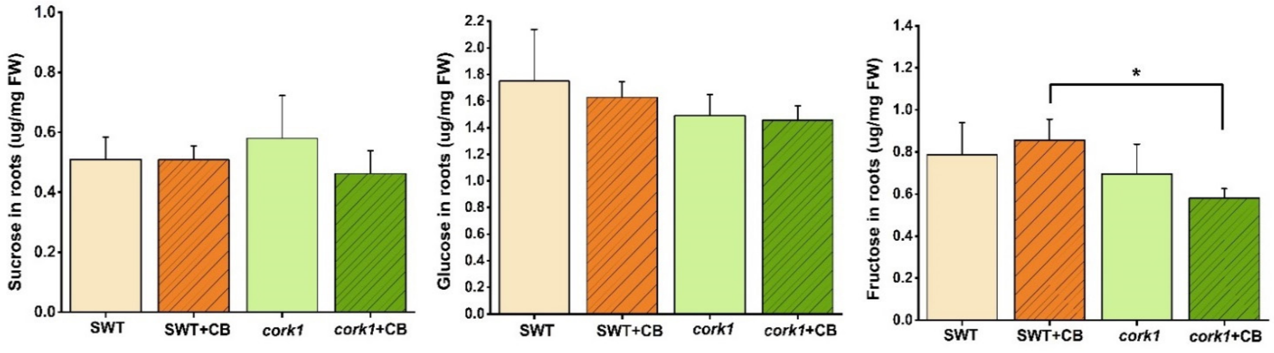

(**B**)

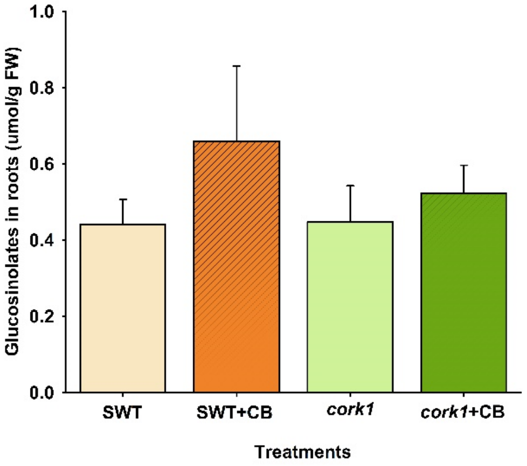

(**C**)

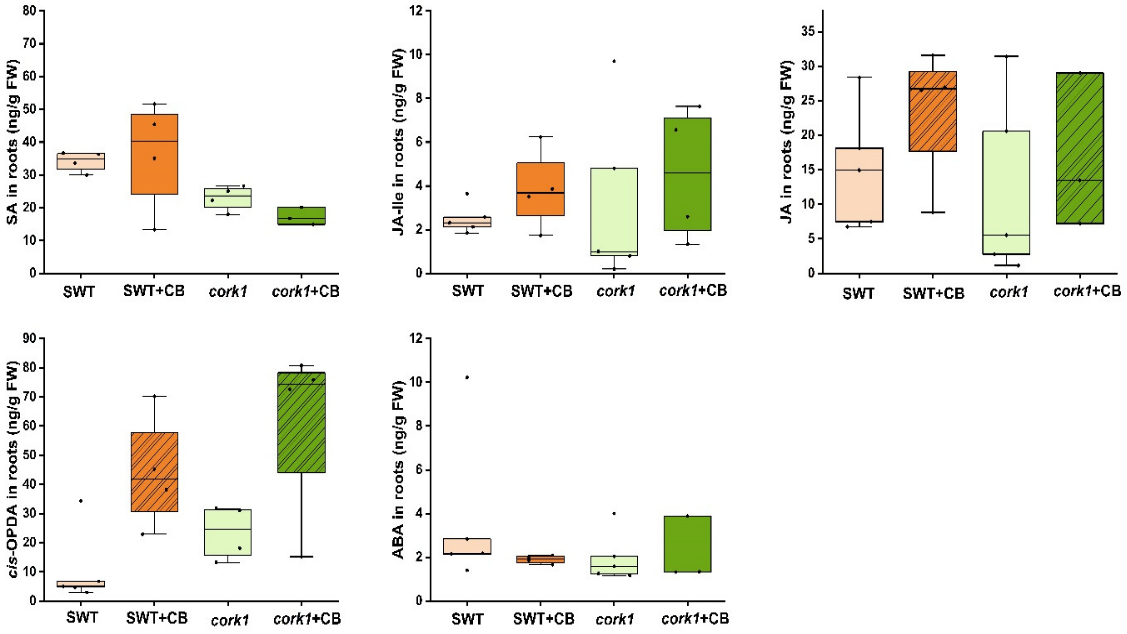

(**D**)

Figure S1. (**A**) Changes in the root fresh weight of SWT (left) and *cork1* seedlings (right) on different concentrations of CB. Significant increase in root weight of wild type seedlings (SWT) grown on 10 mM CB was observed as compared to control without CB. Root weight of *cork1* mutant remained unchanged on different concentrations of CB (right). Asterisks indicate statistically significant differences between groups and control according to Tukey's HSD test (n=20 per treatment; **p*<0.05, ***p*<0.01). (**B**) Soluble sugar contents (sucrose, glucose, fructose) (μg/mg FW) in roots of SWT and *cork1* seedlings grown on media without or with 10 mM CB for 6 days. (**C**) Glucosinolate levels (μmol/g FW) in roots of wild-type and *cork1* seedlings either without or with 10 mM CB. (**D**) Phytohormone levels (SA, salicylic acid; JA-Ile, jasmonoyl-isoleucine conjugate; JA, jasmonic acid; *cis*-OPDA, 12-oxophytodienoic acid; ABA, abscisic acid) (ng/g FW) in roots of 15-day old SWT and *cork1* seedlings either without or with 10 mM CB. SWT and *cork1* mutants were pregrown on half MS media for 9 days and on the 10th day, they were transferred to plant nutrient media either without CB (control) or with filter sterilised CB (1 mM, 10 mM, 30 mM, 50 mM, 100 mM) and grown for additional 6 days. Data are means (± SE) of 5 replicates each consisting of 40-50 seedlings. Statistically significant differences between control (SWT vs *cork1*) and CB-treated (SWT+CB vs *cork1*+CB) were determined according to Student's *t*-test (**p*<0.05, ***p* <0.01). No indication, no significant difference. Shoot weights are not shown as there were no significant differences between treatments.

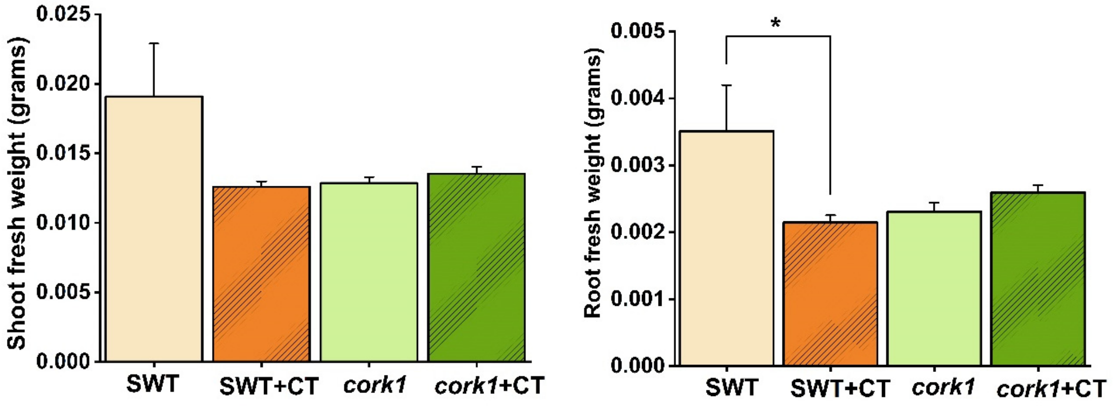
Shoot fresh weight (grams)
0.025
0.020
0.015
0.010
0.005
0.000
SWT
SWT+CT
cork1
cork1+CT
Root fresh weight (grams)
0.005
0.004
0.003
0.002
0.001
0.000
*
SWT
SWT+CT
cork1
cork1+CT

(**A**)

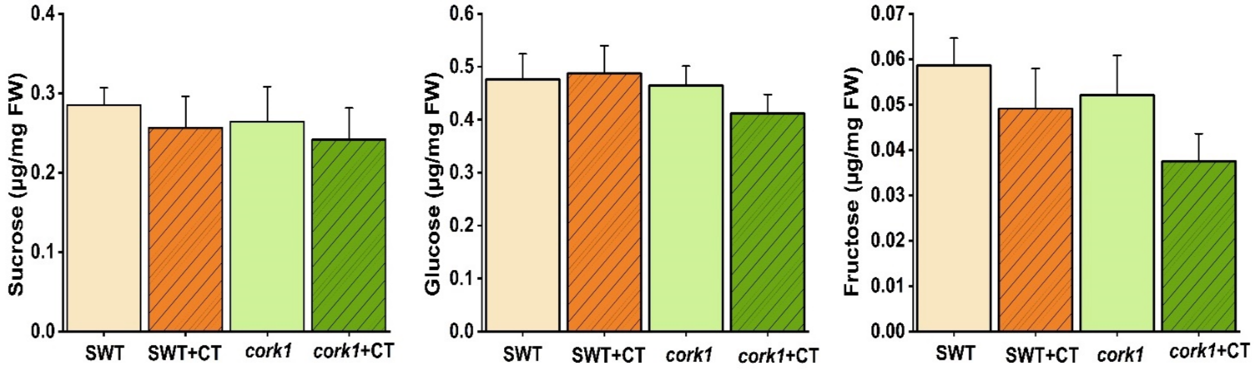
Sucrose (µg/mg FW)
0.4
0.3
0.2
0.1
0.0
SWT
SWT+CT
cork1
cork1+CT
Glucose (µg/mg FW)
0.6
0.5
0.4
0.3
0.2
0.1
0.0
SWT
SWT+CT
cork1
cork1+CT
Fructose (µg/mg FW)
0.07
0.06
0.05
0.04
0.03
0.02
0.01
0.00
SWT
SWT+CT
cork1
cork1+CT

(**B**)

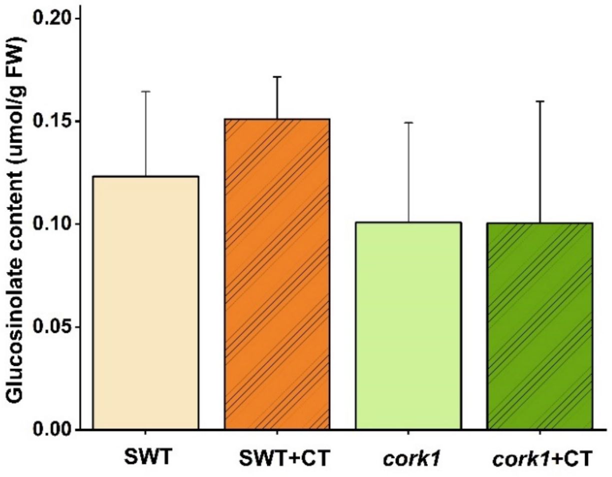
Glucosinolate content (umol/g FW)
0.20
0.15
0.10
0.05
0.00
SWT
SWT+CT
cork1
cork1+CT

(**C**)

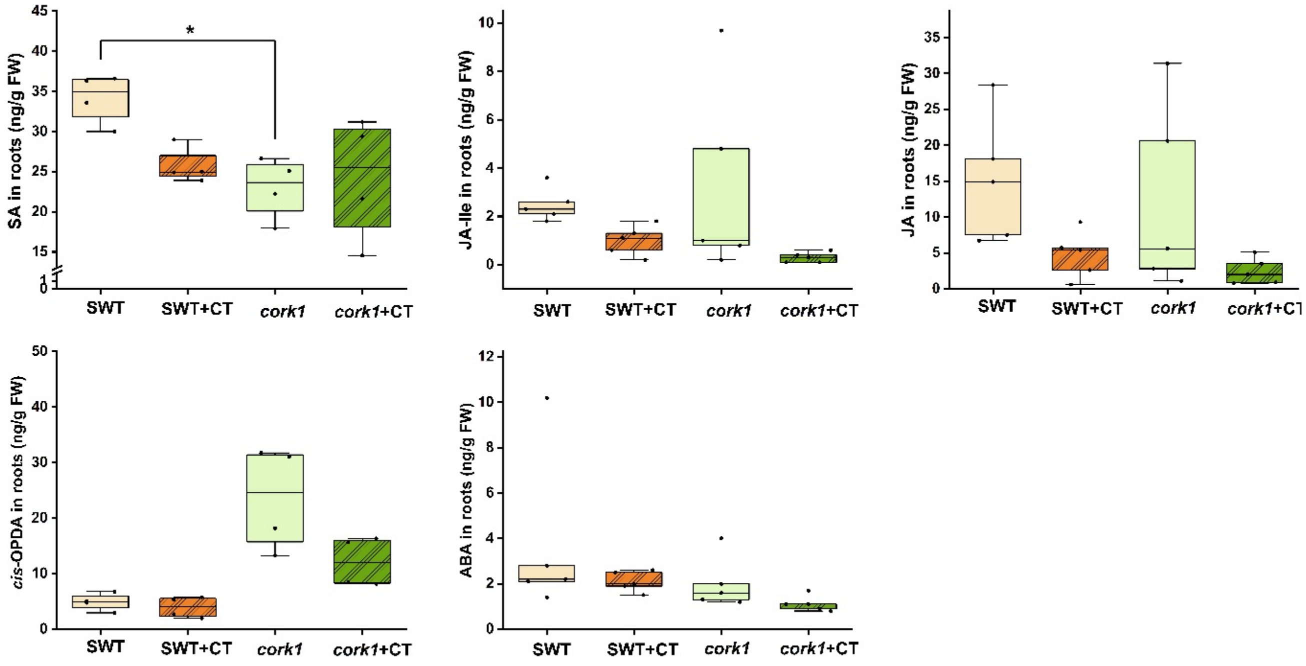

(**D**)

Figure S2. Effect of CT on (**A**) shoot (left) and root (right) fresh weight in SWT and *cork1* mutants. Fifteen-day-old wild-type seedlings display reduced root fresh weight when grown on medium supplemented with 10 µM CT. No differences were observed in the root weight of *cork1* mutants grown on CT. (**B**) Amounts of free sugars (sucrose, glucose and fructose) (µg/mg FW) and (**C**) total glucosinolate levels (µmol/g FW) remain unchanged in roots of both wild-type and *cork1* seedlings treated with CT. (**D**) Phytohormone content (ng/g FW) in roots of SWT and *cork1* plants with or without CT (SA, salicylic acid; JA-Ile, jasmonoyl-isoleucine conjugate; JA, jasmonic acid; *cis*-OPDA, 12-oxophytodienoic acid; ABA, abscisic acid). SWT and *cork1* mutants were grown on half MS media for 9 days and on the 10th day, they were transferred to plant nutrient media either without CT (control) or with 10 µM of filter sterilized CT and grown for additional 6 days. Data are means (± SE) of at least 4 biological replicates each consisting of 40-50 seedlings. Asterisks mark statistically significant differences as determined by one–way ANOVA with Tukey's HSD (* $p \leq 0.05$). No indication, no significant difference.

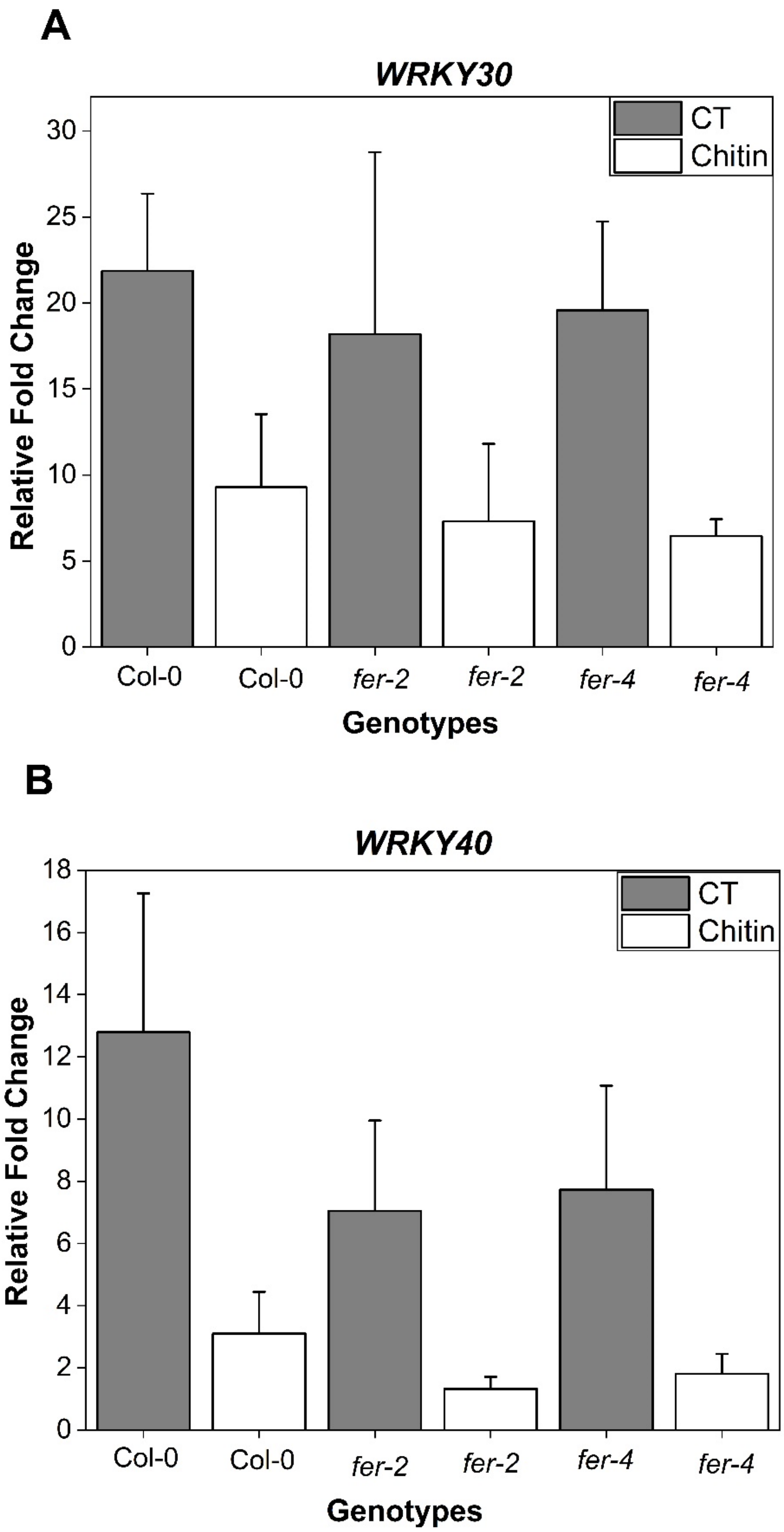

Figure S3. Upregulation of (**A**) *WRKY30* and (**B**) *WRKY40* mRNA levels in root tissue 1 h after treatment with 10 μM CT or 10 μM chitin was not significantly different in wild-type (Col-0), *fer-2* and *fer-4* mutants. Values were normalized to water treatment on the same genotype. Error bars represent SE from 4 independent biological replicates, each with 24 seedlings. Statistical significance between genotypes under the same treatment was determined by Tukey's HSD test with $p \leq 0.05$. No indication, no significant difference.

References

1. Tseng, Y.-H.; Scholz, S.S.; Fliegmann, J.; Krüger, T.; Gandhi, A.; Furch, A.C.; Kniemeyer, O.; Brakhage, A.A.; Oelmüller, R. CORK1, A LRR-Malectin Receptor Kinase, Is Required for Cellooligomer-Induced Responses in *Arabidopsis thaliana*. *Cells* **2022**, *11*, 2960.
2. Gandhi, A.; Tseng, Y.-H.; Oelmüller, R. The damage-associated molecular pattern cellotriose alters the phosphorylation pattern of proteins involved in cellulose synthesis and trans-Golgi trafficking in *Arabidopsis thaliana*. *Plant Signal. Behav.* **2023**, *18*, 2184352.
